# Heterogeneous localisation of membrane proteins in *Staphylococcus aureus*

**DOI:** 10.1038/s41598-018-21750-x

**Published:** 2018-02-26

**Authors:** Felix Weihs, Katarzyna Wacnik, Robert D. Turner, Siân Culley, Ricardo Henriques, Simon J. Foster

**Affiliations:** 1 The Krebs Institute. Department of Molecular Biology and Microbiology, University of Sheffield, Firth Court, Western Bank, Sheffield, S10 2TN UK; 2 Quantitative Imaging and Nanobiophysics Group, MRC Laboratory for Molecular Cell Biology and Department of Cell and Developmental Biology, University College London, Gower Street, London, WC1E 6BT UK; 3 The Francis Crick Institute, 1 Midland Rd, Kings Cross, London, NW1 1AT UK

## Abstract

The bacterial cytoplasmic membrane is the interface between the cell and its environment, with multiple membrane proteins serving its many functions. However, how these proteins are organised to permit optimal physiological processes is largely unknown. Based on our initial findings that 2 phospholipid biosynthetic enzymes (PlsY and CdsA) localise heterogeneously in the membrane of the bacterium *Staphylococcus aureus*, we have analysed the localisation of other key membrane proteins. A range of protein fusions were constructed and used in conjunction with quantitative image analysis. Enzymes involved in phospholipid biosynthesis as well as the lipid raft marker FloT exhibited a heterogeneous localisation pattern. However, the secretion associated SecY protein, was more homogeneously distributed in the membrane. A FRET-based system also identified novel colocalisation between phospholipid biosynthesis enzymes and the respiratory protein CydB revealing a likely larger network of partners. PlsY localisation was found to be dose dependent but not to be affected by membrane lipid composition. Disruption of the activity of the essential cell division organiser FtsZ, using the inhibitor PC190723 led to loss of PlsY localisation, revealing a link to cell division and a possible role for FtsZ in functions not strictly associated with septum formation.

## Introduction

The ability of organisms to grow and proliferate requires the coordination of all aspects of cellular physiology. Bacteria are arguably the simplest form of life without a nucleus and organelles. However, there must be a close coordination of events between the cytoplasm and the cell wall to allow growth and division. The cytoplasmic membrane is the interface between the cytoplasm and the external environment and is required for viability, nutrient acquisition, signalling and myriad other processes. The membrane is made up of a lipid bilayer studded with an array of integral and peripheral proteins along with other biopolymers. How membrane proteins are organised to optimise their function is largely unknown, as are those components involved in such coordination.

In bacteria, prominent systems involved in membrane protein organisation have been studied in detail. Firstly, the actin-homologue MreB acts as a spatial organiser by positioning cell wall synthesis enzymes around the cytoplasmic membrane of *Escherichia coli* allowing maintenance of the characteristic rod-shape^1–6^. In *Bacillus subtilis*, MreB creates membrane regions with increased fluidity which may affect membrane protein diffusion and functionally organise the bacterial membrane^7^. Secondly, the scaffolding protein FtsZ is an early arrival at the division-site forming the Z-ring required for the recruitment of later, cytoplasmic and membrane-associated division proteins^8,9^. The recruited proteins coordinate the synthesis of lipids and peptidoglycan. Finally, the selection of the division-site in many bacteria is determined by further membrane organising components such as the Min proteins, that can use existing geometrical cues in the cell to place the Z-ring at the middle of the cell^10–12^. In both *E. coli* and *B. subtilis* the Min system acts to identify the polar regions to inhibit FtsZ polymerisation in regions other than the mid-cell^10,11^.

The bacterium *Staphylococcus aureus* lacks both MreB and the Min system^13^. Thus, *S. aureus* is a useful model in which to study the properties of membrane proteins and to ask the question: How do bacteria localise membrane proteins in the absence of known organisers?

The study of phospholipid synthesis enzymes in *S. aureus* has led to the discovery of a colocalised punctate patterned distribution of the two membrane proteins PlsY and CdsA^14^. The absence of PlsY causes severe morphological defects and misplaced division septa. Furthermore, the localisation of the septally located cell-division proteins EzrA and PBP2 were affected in PlsY-depleted cells, which may explain the observed aberrant cell-division. It seemed unlikely that PlsY was the key protein for the localisation of this protein network and the focus was directed the MreCD proteins that are known to function as spatial organisers in other bacteria. *S. aureus* lacks MreB and little is known about the role of MreC and MreD^15^. The deletion of *mreC* showed no effect on staphylococcal cells, but an *mreD* null mutant is affected in the localisation of PlsY and CdsA^14^. Further experiments revealed that MreD is also localised in a similar punctate pattern to PlsY and CdsA, showing that MreD might be involved in the establishment of this supramolecular structure^14^. The observed punctate distribution is explicable in terms of the properties of the proteins themselves. The shape of proteins or protein complexes in the membrane can modulate the local curvature and thereby create a local disturbance of diffusion which can result in a patterned distribution of membrane proteins^14^.

This study aimed to identify components required for the observed membrane protein distribution and to test the effect of a variety of inhibitors and genetic interventions. Furthermore, the distribution of a range of membrane proteins was determined to understand the breadth of that phenomenon.

## Results

### Enzymes involved in phospholipid synthesis are distributed heterogeneously in the membrane of *S. aureus*

**Figure 1:**
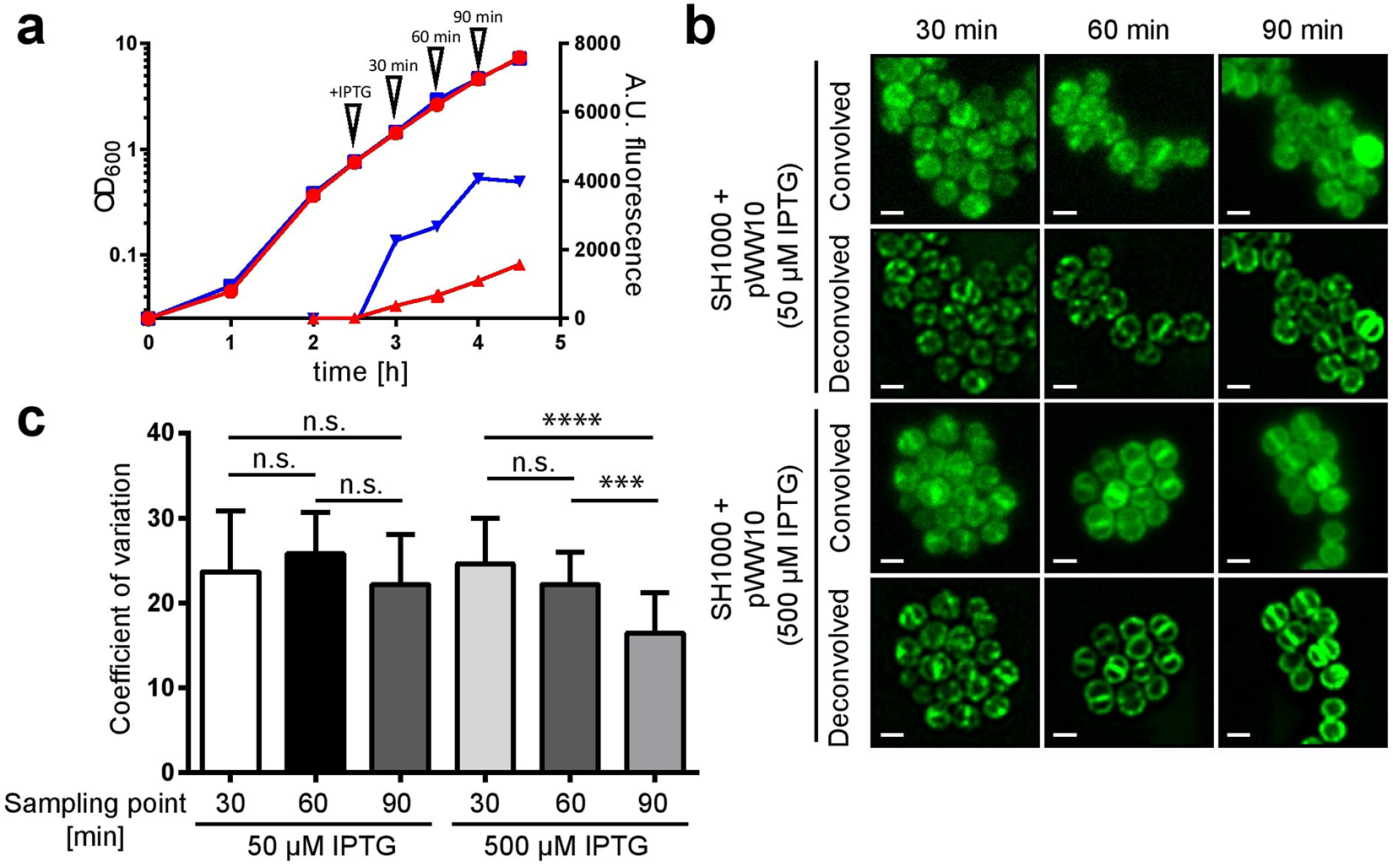
The localisation of PlsY-GFP is dose-dependent. (**a**) Growth curves (left Y-axis) of *S. aureus* SH1000 FW6 (IPTG-inducible *plsY-gfp* expression) and fluorescence (right Y-axis) of whole culture samples. 50 or 500 µM IPTG were added to cultures at an OD_600_ ~ 1 and samples were analysed at 30 min intervals. A.U. fluorescence for 50 (**)** and 500 (**)** µM IPTG induction. OD_600_ for 50 (**)** and 500 (**)** µM IPTG induction. (**b**) Fluorescence images (convolved and deconvolved) of *S. aureus* SH1000 FW6 with different expression levels of *plsY-gfp* as seen in (**a**). Scale bars represent 1 µm. (**c**) CV-factor calculation of deconvolved fluorescence images of PlsY-GFP (FW6). Significance values were calculated using a two-tailed unpaired student t-test. ****P < 0.0001; ***P < 0.001.

PlsY is localised heterogeneously in the membrane of *S. aureus* forming clearly observable puncta of accumulated molecules (Fig. S1a). Counterstaining with the fluorescent D-amino acid HADA (7-hydroxycoumarin-3-carboxylic acid-amino-D-alanine) that labels peptidoglycan synthesis^16^, shows that PlsY localises to the septum in cells undergoing cell-division, with an additional single dot at its centre in cells with a seemingly completed septum. This localisation can be most clearly observed in exponentially growing cells and diminishes as cells enter stationary phase (Fig. S1b,c) which was confirmed using the super-resolution approach NanoJ-SRRF^17^ (Fig. S1d,e). Importantly, PlsY localises in a dose-dependent manner (Fig. 1a) where over-expression leads to a more uniform distribution of fluorescence signal (Fig. 1b,c). To analyse, often subtle, localisation changes and to discriminate whether the fusions are localised homogeneously or heterogeneously required a quantitative image analysis method. This was achieved by the conversion of an image of a cell to a polar coordinate system with the origin at the centre of the cell, followed by calculating the coefficient of variation (CV; CV = mean value/standard deviation). The CV describes the distribution of fluorescence signal around the cell periphery, where low values indicate a more homogeneous distribution and higher values a heterogeneous distribution (see Fig. S2a,b for examples and controls). This analysis confirms that over-expression of PlsY-GFP leads to the collapse of the punctate patterned localisation.

The striking punctate pattern of PlsY begs the question of whether other enzymes involved in phospholipid synthesis (apart from CdsA) are localised in a similar fashion? An overview of phospholipid synthesis in *S. aureus* can be found in Fig. S3.

Fusions of eYFP with PlsY, the phosphatidylglycerol-phosphate synthase PgsA and the major cardiolipin synthase Cls2 were constructed in *S. aureus* SH1000 and analysed by fluorescence microscopy. Western blotting confirmed the expression of all single-copy native promoter fusions used in this study (Fig. S4a).

**Figure 2:**
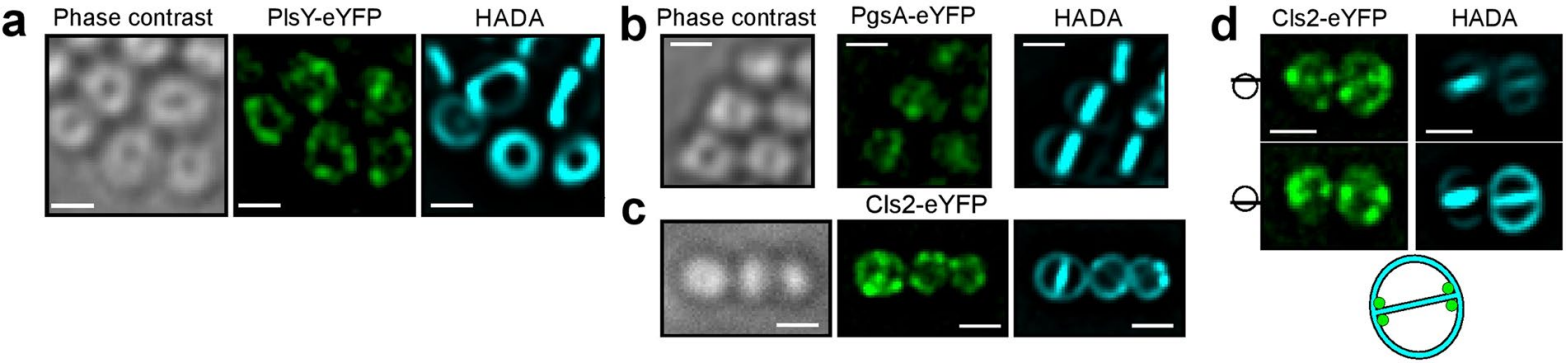
Phospholipid synthesis enzymes are distributed heterogeneously in the membrane of *S*. *aureus*. (**a**–**c**) Phase contrast and fluorescence images (deconvolved) of *S. aureus* expressing *plsY-eyfp* (FW1), *pgsA-eyfp* (FW2) and *cls2-eyfp* (FW5) under their native promoter. Cells were counterstained with the fluorescent D-amino acid HADA for 5 min, which is incorporated into the cell wall indicating the cell-cycle stage. (**d**) Fluorescence images (deconvolved) of Cls2-eYFP in *S. aureus* SH1000 FW5 at the upper and lower end of the cell focus showing the localisation of Cls2-eYFP on different three-dimensional levels. The cartoon schematically illustrates the distribution of Cls2-eYFP at the base of the septum. All scale bars represent 1 µm.

All fusions were found to be localised at the septum in cells undergoing cell division along with a non-homogeneous distribution at the cell periphery (Fig. 2a–d). Interestingly, Cls2-eYFP exhibits a clear punctate distribution and appears to show some septal localisation (Fig. 2d). However, the same cells also show a non-septal punctate Cls2 localisation.

Previous studies have reported that the eYFP tag can cause artifactual localisations of the extended protein of interest possibly due to the multimerisation of eYFP^18,19^. A fusion of PlsY with the monomeric meYFP (A206K)^20^ was also found to exhibit a heterogeneous distribution (Fig. S1f). This confirms that the localisation of PlsY-eYFP is not caused by the potential multimerisation of the fluorescent tag.

### A broader perspective on the localisation of membrane proteins

**Figure 3:**
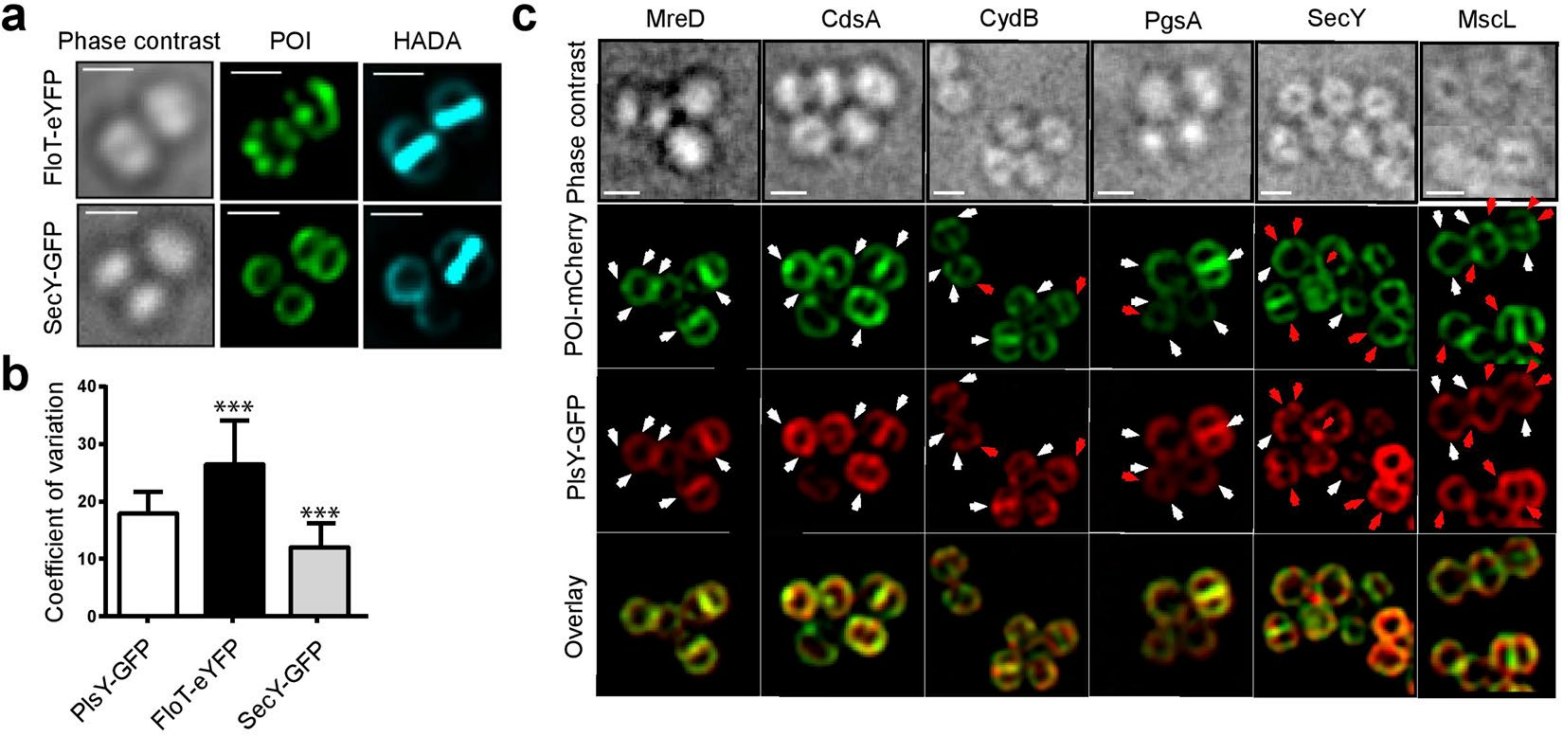
Membrane proteins in *S. aureus* show different localisation profiles. (**a**) Phase contrast and fluorescence images (deconvolved) of *S. aureus* SH1000 expressing *floT-eyfp* (FW8) or *secY-gfp* (JGL231) under their native promoter. Cells were counterstained with HADA for 5 min. (**b**) CV-factor calculation of deconvolved images of PlsY-GFP (JGL232), FloT-eYFP (FW8) and SecY-GFP (JGL231). Significance values were calculated against PlsY-GFP using a two-tailed unpaired student t-test. ***P < 0.001. (**c**) Colocalisation studies of PlsY-GFP with a range of membrane proteins translationally fused to mCherry in *S. aureus* RN4220 (strains FW14-FW20). Fusions were expressed from an IPTG-inducible plasmid and fluorescence images were deconvolved. White arrows indicate matching foci of fluorescence signals while red arrows show non-matching signal foci. All scale bars represent 1 µm.

Phospholipid synthesis enzymes exhibit a heterogeneous distribution along with a septal localisation during cell division. To determine if this is a more generalised phenomenon, the localisation of other membrane proteins from metabolic processes unrelated to phospholipid synthesis was tested. The recently identified lipid raft marker FloT^21^ and the secretion associated protein SecY were chosen. First, protein fusions with eYFP or GFP expressed from their native loci (Fig. S4a) were analysed and revealed a punctate patterned distribution of FloT whereas the secretion protein SecY appears to be localised homogeneously (Fig. 3a). CV calculations confirm this finding and further indicate that FloT has a more distinct distribution of fluorescence signal according to the CV-factor. Such a defined localisation of FloT-eYFP is supported by previous studies^21^. SecY exhibits a significantly lower CV-factor than PlsY supporting a more homogeneous distribution (Fig. 3b). It must be considered that the observed punctate distribution of these membrane proteins could be in fact part of a, possible cell-spanning, three-dimensional structure, as a Z-stack image series of PlsY-GFP shows that the puncta are found all around the cell periphery (Fig. S5).

**Figure 4:**
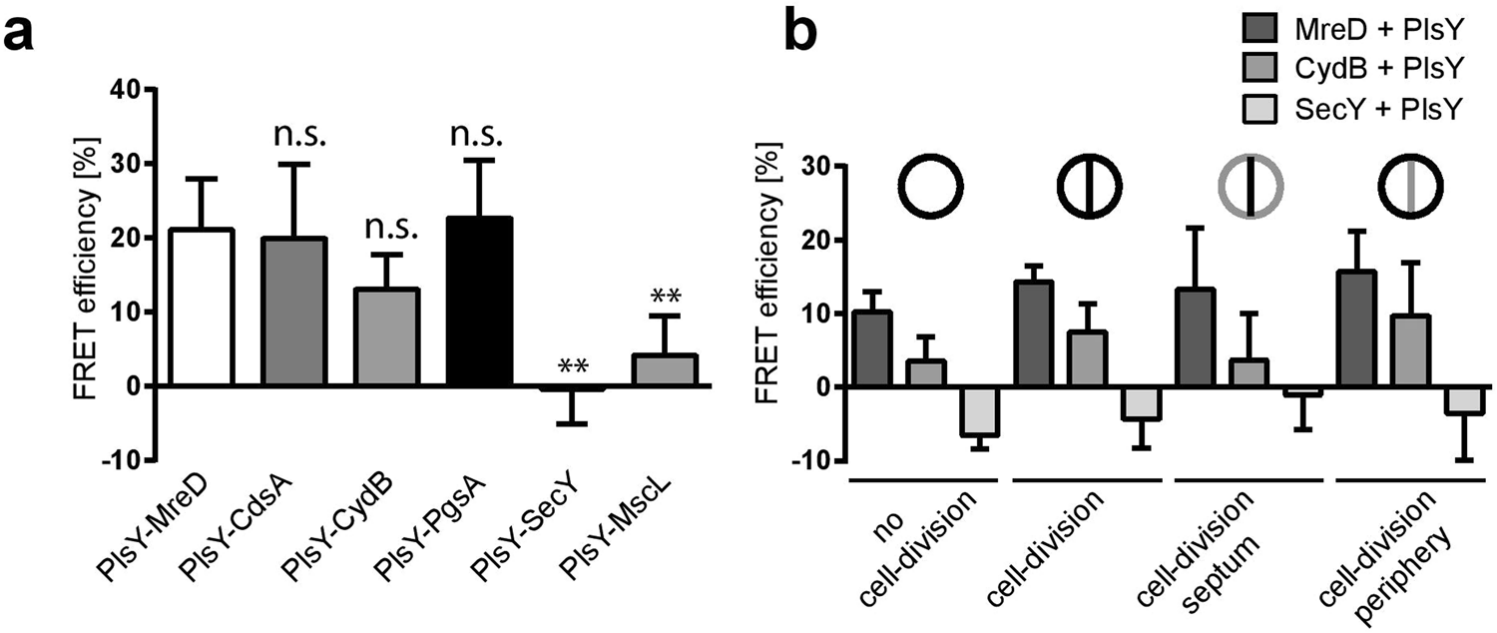
Phospholipid synthesis enzymes, MreD and CydB interact with PlsY. (**a**) FRET efficiencies calculated using a donor photo bleaching FRET system. All investigated strains expressed *plsY-gfp* together with a protein of interest translationally fused to *mCherry* (strains FW14-FW20). Significance values were calculated using a two-tailed unpaired student t-test. **P < 0.01. The interaction analyses of PlsY with MreD, CdsA, SecY and MscL were shown previously^14^. (**b**) FRET efficiencies of protein interactions on a subcellular level of PlsY with MreD, CydB or SecY. Non-dividing and dividing cells were analysed. In addition, dividing cells were further dissected into the septum and periphery. In the case of interactions with SecY some negative FRET efficiency values were calculated. We do not, of course, claim a true negative FRET efficiency, simply that the donor fluorophore bleached more rapidly in these experiments. It was necessary to include these results for completeness.

To begin to determine if particular proteins form parts of complexes, colocalisation studies with PlsY-GFP were carried out. Fusions of various membrane proteins with mCherry were expressed episomally from an IPTG-inducible promoter (Fig. 3c) and their expression was confirmed by Western blotting (Fig. 4b). As expected, the membrane proteins are localised in a heterogeneous distribution. SecY-mCherry was localised in a punctate pattern, which could indicate that the expression level is lower than native levels having a similar dose-dependent effect as PlsY (Fig. 1). In this analysis, we included the membrane protein MscL (mechano sensitive channel protein) and the respiratory protein CydB, which also exhibit a non-uniform distribution (Fig. 3c). Image analyses suggested colocalisation (see white arrows for colocalising dots and red arrows for differing localisation) and demonstrated that PlsY is colocalised with MreD, CdsA, PgsA and potentially CydB, with no or less colocalisation with SecY and MscL. A quantitative pixel-by-pixel analysis using the Manders overlap coefficient^22^ between ‘green’ and ‘red’ fluorescence signals confirmed these observations demonstrating a significantly higher colocalisation of ‘green’ pixels (from PlsY-GFP) with signal derived from mCherry fusions with MreD, CdsA, PgsA and CydB (Manders values of ~0.7–0.8) than with SecY and MscL (Fig. S2c) (Manders values of ~0.6). Thus, membrane protein distribution in *S. aureus* is often heterogeneous but with a diversity of arrangements.

### Evidence for a phospholipid synthesis enzyme complex

Recently, we showed the interaction of PlsY with CdsA and MreD using a protein-protein interaction system based on Förster Resonance Energy Transfer (FRET) facilitating lower donor photobleaching rates of GFP in the presence of mCherry^14^. This FRET method is simple in terms of image acquisition using a standard widefield microscope. The analysis was extended to investigate whether other membrane proteins that exhibit a punctate patterned distribution such as PgsA and CydB interact with PlsY. In addition, the interaction of PlsY with the non-co-localising MscL and SecY was studied. PlsY-GFP was used as the donor in all experiments as this is the most characterised of the fusions and gave consistency with all the other protein fusions.

This analysis corroborates the previous findings that PlsY is colocalised with PgsA and CydB, as a positive interaction was found with both proteins (FRET efficiency (FE): 22.7% for PgsA and FE: 13.0% for CydB) but not with SecY (FE: −0.3%) and MscL (FE: 4.1%) of which were shown to be localised in a different pattern than that of PlsY (Fig. 4a).

The FRET-based protein-protein interaction system also allowed subcellular determination of protein co-localisations. This was achieved by the analysis of GFP fluorescence intensity over time of selected subcellular compartments such as cells that are or are not undergoing cell division and the cell periphery or septum of dividing cells. Interactions were found in cells undergoing cell division as well as in cells without obvious septa. CydB interacts with PlsY mainly in dividing cells at the cell-periphery while the interaction of MreD with PlsY occurs at all analysed subcellular levels (Fig. 4b). Consistent with the whole cell FRET analysis, PlsY does not interact with SecY in any subcellular compartment. Overall, these interaction analyses suggest the formation of a phospholipid synthesis enzyme complex consisting of at least of PlsY, CdsA and PgsA which would allow possible metabolic channelling of intermediates. MreD might act as a spatial organiser to stabilise the complex formation as we showed previously^14^. The involvement of CydB in this complex suggests a higher level of complex formation linking several cellular aspects.

### MreD of *S. aureus* exhibits a non-uniform distribution when expressed heterogeneously in *E. coli*

**Figure 5:**
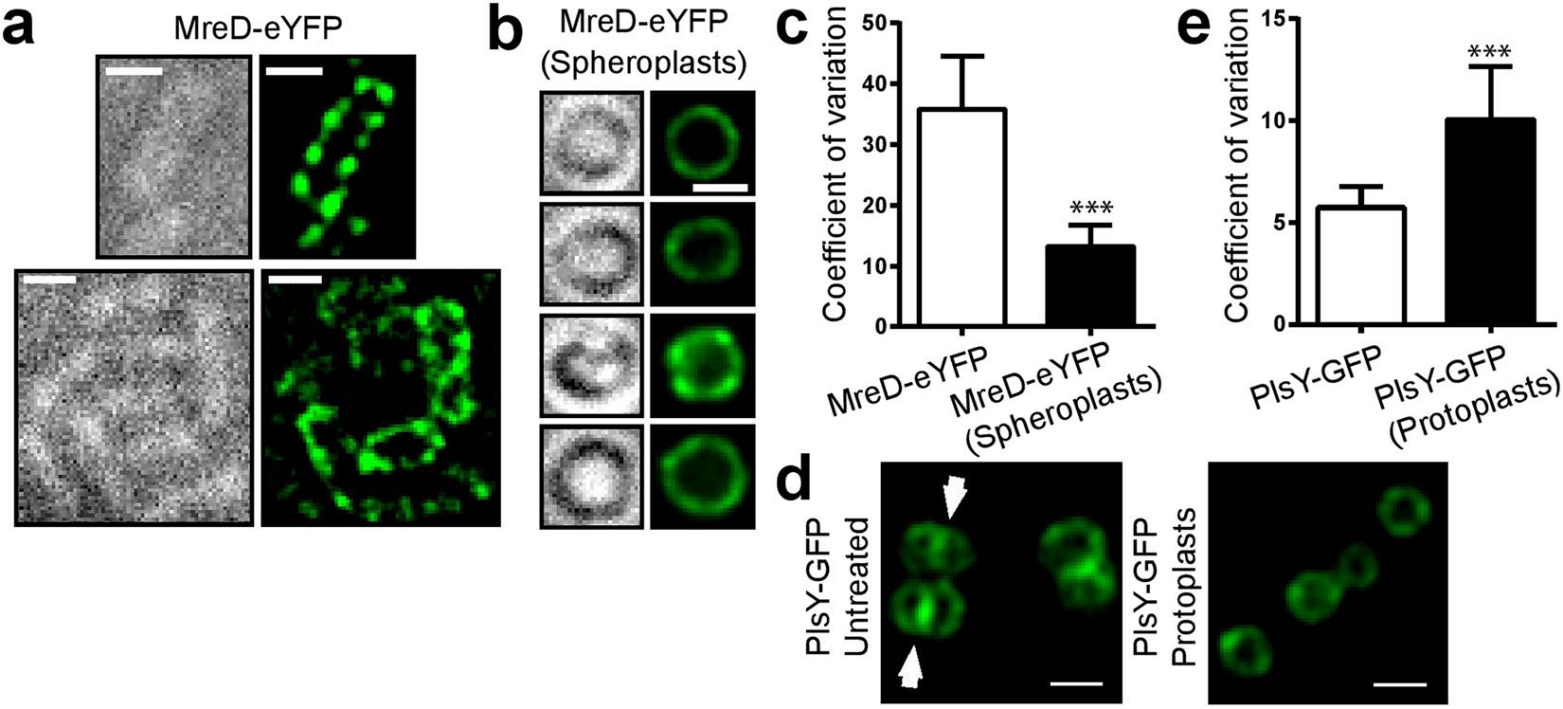
Role of the cell wall in membrane protein localization. (**a**) Phase contrast and fluorescence images (deconvolved) of *E. coli* C43(DE3) with episomal IPTG-induced expression of *mreD-eyfp* (*E. coli* C43(DE3) *mreD-eyfp*). (**b**) Phase contrast and fluorescence images (deconvolved) of spheroplasts of *E. coli* C43(DE3) *mreD-eyfp*. (**c**) CV-factor calculation of deconvolved images of MreD-eYFP in *E. coli* C43(DE3) *mreD-eyfp* rods and spheroplasts. (**d**) Fluorescence images (deconvolved) of protoplasted and native cells of *S. aureus* SH1000 JGL232 (*plsY-gfp*). White arrows indicate septal PlsY-GFP localisation, which is not seen in protoplasts. (**e**) CV-factor calculation of deconvolved images of PlsY-GFP in native cells and protoplasts of *S. aureus* SH1000 JGL232 (*plsY-gfp*) (based on 10 cells for each group). All significance values were calculated using a two-tailed unpaired student t-test. ***P < 0.001. All scale bars represent 1 µm.

If heterogeneous pattern formation is an intrinsic property of the proteins, then this might also be propagated when expressed in a heterologous host. Expression of PlsY-GFP in *E. coli* was found to be lethal leading to cell lysis (data not shown). Expression of *S. aureus* MreD fused to eYFP in *E. coli* revealed a striking punctate patterned distribution, where MreD appears to be localised in foci along the membrane of the cell cylinder and it does not preferentially accumulate at the poles (Fig. 5a). The role of the cell wall in the localisation of MreD was determined by spheroplast generation of the fusion containing *E. coli* strain by treatment with lysozyme and addition of EDTA to the medium. Interestingly, MreD-eYFP localisation was altered upon spheroplast formation and the fusion was more homogeneously dispersed in the membrane as shown by microscopy (Fig. 5b) and CV-calculations (Fig. 5c, see Fig. S2d for MreD-eYFP in rod-shaped *E. coli*). Thus, potentially cell geometry is needed for MreD pattern formation in *E. coli*, or alternatively a functional cell wall might be required. Of course, expression in a heterologous host is an artificial situation and needs to be interpreted with caution. The role of a functional cell wall was tested for PlsY-GFP in *S. aureus* by protoplast formation after lysostaphin digestion. Both, lysostaphin treated and untreated (control) cells were suspended in a sucrose buffer that prevents protoplasts lysis. Image analysis revealed that PlsY-GFP in protoplasts is more heterogeneously distributed than in untreated cells (Fig. 5e). Protoplasts do not display septa and so this localisation of PlsY is lost (Fig. 5d). The redistribution of PlsY from the septum to the cell periphery during protoplast formation might create a more distinct heterogeneous localisation pattern and also indicates that the underlying mechanism that distributes PlsY is not found at the cell wall.

### Understanding the basis of membrane protein distribution

To begin to understand those mechanisms that could underpin the observed protein patterns, with the discovery of domains and lipid rafts it has become evident that the bacterial membrane is highly organised with specific lipids and associated proteins forming specialised subcellular compartments. Squalene-dependent lipid rafts^21,23,24^, cardiolipin^25–29^ or phosphatidyl-ethanolamine membrane domains^30,31^ were reported to preferentially localise to certain sub-cellular cues or distribute heterogeneously in discrete foci.

**Figure 6:**
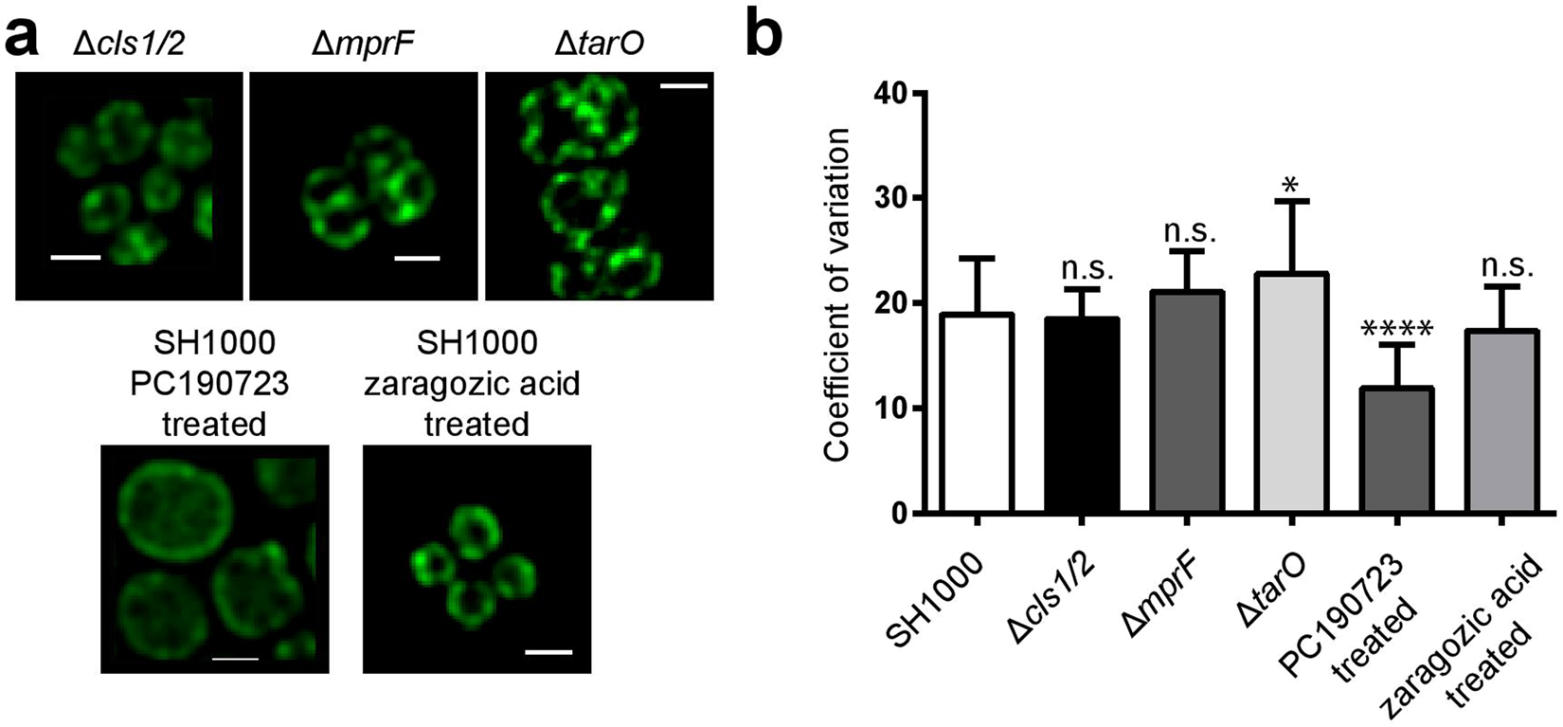
Inhibition of FtsZ disrupts the localisation pattern of PlsY. (**a**) Fluorescence images (deconvolved) of: FW21, *S. aureus* SH1000 *plsY-eyfp* in CL-deficient background (∆*cls1/2*); FW22, *S. aureus* SH1000 *plsY-eyfp* in LPG-deficient background (∆*mprF*): FW23, *S. aureus* SH1000 *plsY-eyfp* in WTA-deficient background (∆*tarO*); JGL232 (*S. aureus* SH1000 *plsY-eyfp*) treated with the FtsZ inhibitor PC190723 and the squalene-synthase inhibitor zaragozic acid. Scale bars represent 1 µm. (**b**) CV-factor calculation of deconvolved images of all investigated groups. Significance values against the untreated group were calculated using a two-tailed unpaired student t-test. ****P < 0.0001; *P < 0.05.

The staphylococcal membrane is mainly composed of three phospholipids: phosphatidyl-glycerolphosphate (PG), lysinylated phosphatidylglycerolphosphate (LPG) and cardiolipin (CL)^32,33^. LPG is synthesised by MprF, PG by PgsA and cardiolipin requires the enzymes Cls1 and Cls2. The *pgsA* gene is apparently essential^34^, but *mprF*, *cls1* and *cls2* have been previously characterised genetically^35–37^. Using *S. aureus* SH1000 strains carrying the respective mutations both LPG and CL were found to have no apparent role in the localisation of PlsY-GFP by CV analysis (Fig. 6a,b).

Lipid rafts in *S. aureus* have been described to require the cholesterol precursor squalene^21^. The synthesis of squalene can be inhibited by zaragozic acid, which in turn causes the degradation of lipid raft cargo such as FloT. Although we could observe the degradation of FloT-eYFP by a loss of fluorescence when cells are treated with zaragozic acid, no effect on the localisation of PlsY-GFP could be seen (Fig. 6a,b and Fig. S6) suggesting that squalene is not required for the positioning of PlsY.

To test the role of cell metabolic status in PlsY localisation, cells were treated with the fatty acid synthesis inhibitor cerulenin^38^ and the uncoupling agent carbonyl cyanide m-chlorophenyl hydrazone (CCCP)^39^. CCCP has been demonstrated previously to cause the delocalisation of MreB in *B. subtilis*^40^. In both cases, even though growth of *S. aureus* was inhibited, the PlsY-localisation was unaffected (Fig. S7a,b).

SDS is an ionic detergent that denatures proteins and treatment of cells led to a loss of the PlsY distribution increasing its CV value from 15.9 in untreated cells to 24.7 in cells treated with 2 mM SDS (Fig. S8c). The punctate distribution of PlsY disappeared and it was found in one or two patches in the membrane (Fig. S8a,b). As SDS intercalates into the membrane, it may perturb and alter the overall integrity and local geometry required for the localisation of PlsY.

Wall teichoic acids (WTAs) are polymers covalently attached to the cell wall peptidoglycan and have been shown to be important for the localisation of the cell wall associated amidase Atl^41^ and of the cell wall biosynthetic proteins PBP4 and Fmt^42,43^. The *tarO* gene is required at an early stage in WTA biosynthesis and its deletion led to a substantially greater CV value for PlsY-GFP (Fig. 6a,b). The *tarO* cells also have a size increase (Fig. S9) compared to their parent, which may result in more clearly observable PlsY-foci.

The CV value for the *tarO* mutant was increased concomitant with a greater cell volume (Fig. 6a,b and Fig. S9). To determine if the cell size is a contributor to PlsY localisation, cells were treated with the FtsZ inhibitor PC190723^44,45^. The inhibitor stabilizes FtsZ protofilaments into foci, preventing cell division^44,45^. It causes staphylococcal cells to ‘swell’^44^, as fluorescent amino acid incorporation studies show peptidoglycan is laid down around the cell instead of mostly at the septum^46^. Surprisingly, cells treated with PC190723 exhibited a more homogeneous distribution of PlsY (Fig. 6a,b and Fig. S10a,b,c). The CV-factor dropped from 19 (untreated) to 13 (60 min treatment) and remained at that level while cells continuously increase in size (Fig. S10b,c). Thus, no correlation between CV-factor and cell size is observed.

## Discussion

An analysis of membrane protein distribution revealed a heterogeneity in the membrane of *S. aureus*. This is manifested as a punctate pattern that coalesces at the septum during division, with proteins also present around the rest of the cellular periphery. A key question is to explain how such heterogeneous distributions arise, are maintained and change during the cell cycle?

Theoretically, membrane protein patterns such as the ones observed here can be generated by the intrinsic properties of the molecules themselves^14^. If integral membrane protein complexes impose a sufficiently large local curvature on the membrane, protein complexes can themselves spontaneously form the observed patterns. A homogeneous or random distribution would be accompanied with high energy costs to counteract the intrinsic locally induced membrane curvature imposed by the protein complexes. This model provides a basic framework from which to test the diversity of localisation behaviours and those many parameters that determine the observed output.

Inhibition of FtsZ by PC190723 caused the disruption of PlsY-localisation. This is not simply due to loss of septation as *S. aureus* protoplasts still exhibit the PlsY localisation pattern. FtsZ is well known to mediate cell division but also has a function in cell elongation in rod-shaped cells^47,48^. This suggests that FtsZ has a hitherto undescribed role in membrane protein organisation, independent of septation in *S. aureus*. This may occur directly or via another factor that acts to control protein distribution. The action of PC190723 is also important as it reaffirms the heterogeneity in PlsY localisation in the untreated cells.

A redistribution of *S. aureus* MreD (MreD-eYFP) in *E. coli* also occurred in spheroplasts resulting in loss of its heterogeneous distribution. During heterologous expression, SaMreD might be localised within the MreBCD complex^49,50^ and be affected by the changed shape leading to its delocalisation.

A previous study described a link between the acyl–acyl carrier protein phosphate acyltransferase PlsX in *Bacillus subtilis* with the cell divisome^51^. PlsX was found to localise in a punctate pattern and its deactivation caused aberrant Z-ring formations reminiscent of the role of PlsY in *S. aureus*^14^. In addition, Takada *et al*., proposed that PlsX localises prior to the Z-ring at the future division site, a claim supported by localisation studies and an interaction found between PlsX and the FtsZ-anchoring protein FtsA^51^. It seems likely that PlsY, potentially together with PlsX, has a similar role in *S. aureus* and may contribute to the future cell division site placement with an interplay directly or indirectly with FtsZ. A very recent study demonstrated that PlsX in *B. subtilis* is localised in fluid membrane micro domains that are targeted by daptomycin^52^. This finding fits into our observations in *S. aureus* for the localisation of other phospholipid synthesis enzymes suggesting that fluid membrane micro domains may also be found in *S. aureus*. A logical next step would be to investigate whether the staphylococcal membrane is composed of differentially fluid membrane compartments and their relation to heterogeneously localised membrane proteins.

The cytochrome BD subunit II CydB localises in patches mainly at the cell-periphery. This is consistent with findings in *E. coli* describing CydB to be concentrated in mobile domains^53^. Other studies on respiratory proteins such as the succinate dehydrogenase (SDH) and ATP synthase in *B. subtilis*^54^ or the SDH and the NADPH dehydrogenase in *Synechococcus elongatus* revealed a localisation pattern in discrete spots in the membrane^55^. More evidence for a laterally heterogeneous membrane organisation was shown in *Synechocystis sp*. PCC 6083 where all four FtsH proteases, which play an important role in the repair of photo-damaged photosystem II, show distinctively patchy distributions in the membranes^56^.

Here we found an apparent homogeneous non-septal localisation for SecY, as has been described for *E. coli*^57^ and *B. subtilis*^58^. However, another study using fluorescently labelled secretion substrate coupled with SecY and SecA GFP-fusions demonstrated that secretion takes place at discrete foci along the tubular axis of *B. subtilis*^59^. Perhaps this highlights a diversity of localisation mechanisms across the species.

Under the control of its native promoter, PlsY, and other phospholipid biosynthetic proteins exhibit a heterogeneous distribution in the membrane. PlsY-GFP puncta were only observed when weakly expressed from an IPTG-inducible system and higher expression led to uniformly distributed PlsY. A similar observation was made by Nenninger *et al*., analysing a model membrane protein in *E. coli*, which distributes in domains of about 100 nm at low concentrations while the localisation pattern was lost at higher expression levels^60^. It was suggested that increased levels of protein will result in too high number of these domains to be able to distinctively visualise them. Thus, the level of membrane protein affects the distribution.

Collectively, our observations are in agreement with the ‘compartmentalised fluid’ or ‘partitioned’ model of biological membranes^61–63^. Accordingly, a random membrane protein distribution must be regarded as the exception rather than the rule. The evolution of patterning as part of intrinsic protein properties pertains to optimisation of function. Thus, metabolic channelling through the formation of protein complexes anchored to the membrane through the bending imposed by the complexes themselves could be a common mechanism. The supramolecular organisation of membrane proteins described here and in other recent studies^14,53,55,56,60^ could be a common feature and apply across all biology.

## Methods

### Bacterial strains and growth conditions

All strains used in this study are listed in Supplementary Table 1. *E. coli* was grown in lysogeny broth (LB) and *S. aureus* in brain heart infusion (BHI) medium at 37 °C. Medium was supplemented with the following antibiotics when applicable: Ampicillin (Amp) 100 μg/ml; Kanamycin (Kan) 100 μg/ml; Erythromycin (Ery) 5 μg/ml; Lincomycin 25 μg/ml; Tetracycline (Tet) 5 μg/ml; Chloramphenicol (Cm) 10 μg/ml.

Labelling of peptidoglycan synthesis was achieved by incubation of cell cultures in 7-hydroxycoumarin-3-carboxylic acid-amino-D-alanine (HADA^46^; 50 µM) at 37 °C for 5 min or 30 min (for PC190723 treated cells).

SDS treatment of cell was performed on 1 ml bacterial culture samples of exponentially growing cells that were harvested by centrifugation and resuspended in PBS supplemented with various concentrations of SDS (0, 0.125, 0.25, 0.5, and 2 mM). These were incubated covered in foil for 10 min at RT on a rotary wheel before analysis by fluorescence microscopy.

The effect of cerulenin (100 µM), carbonyl cyanide m-chlorophenyl hydrazone (CCCP; 0.5 and 2.5 µm) and zaragozic acid (1 and 10 µM) on the localisation of PlsY-GFP was tested by growth in the presence of the inhibitor. All chemicals were added to the growth medium after dilution of an overnight culture while the FtsZ inhibitor PC190723 (10 µg/ml) was added after the cell culture reached on OD_600_ ~ 0.5. Cells were analysed prior to and after 60, 90 and 120 min of addition of PC190723. Additionally, to indicate the cell cycle stage, cells were labelled with HADA (50 µM) during the final 30 min of PC190723 treatment.

### Protoplast generation of *S. aureus*

*S. aureus* SH1000 *plsY-gfp* was subcultured from an over-night culture to an OD_600_ = 0.05 and grown in BHI medium for 3 h to an OD_600_≈1.5. Cells were recovered by centrifugation and resuspended in SMM-BHI medium (50% BHI (v/v), 50% SMM (v/v) (SMM: 1 M sucrose, 0.04 M maleic acid, 0.04 M MgCl_2_ × 6H_2_O, pH 6.5)). The culture was then split into two 500 μl fractions. One fraction was treated with 5 μl lysostaphin (5 mg/ml stock solution) for 10 min at RT on a rotary wheel whereas the other fraction was treated the same way without lysostaphin. Protoplast generation was monitored by loss of turbidity and CFU when treated with 1% (w/v) SDS. Protoplasts were placed on a non-coated glass slide and analysed by fluorescence microscopy

### Spheroplast generation of *E. coli*

Overnight-cultures of *E. coli* C43(DE3) *mreD-eyfp* (*E. coli* C43(DE3) containing the plasmid pWALDO-*mreD-eyfp*) were diluted in BHI to an OD_600_ = 0.05 and grown at 37 °C for 1 h in the presence of kanamycin (50 µg/ml), 250 rpm. Subsequently, 0.6 mM IPTG was added and cultures were grown for another 2 h at 37 °C. Cells were harvested and washed with ice-cold Tris-HCl (10 mM, pH 7.5) and the pellet was resuspended to an OD_600_ = 0.6 in sucrose buffer (33 mM Tris-HCl (pH 8.0), 20% sucrose (w/v)). 80 µl of EDTA (0.1 M) and 400 µl lysozyme (1 mg/ml) were added to 1 ml of resuspended cells and the tube was covered in foil and incubated at 4 °C on a rotary wheel for 30 min. Cells were then washed with and resuspended in ice-cold sucrose buffer. Prepared spheroplasts were placed on non-coated glass slides and analysed by fluorescence microscopy.

### Strain and plasmid construction

Oligonucleotides were manufactured by Eurofins Genomics (Ebersberg, Germany) and DNA sequences were verified by Sanger DNA sequencing services provided by GATC Biotech AG (Konstanz, Germany). All oligonucleotides used in this study are listed in Supplementary Table 2. A detailed description of the strain construction procedure can be found in the Supplementary Information.

### Western blotting

Western Blotting Substrate was purchased from BioRad. A detailed description of the western blotting procedure can be found in the Supplementary Information.

### Fluorescence microscopy

Unless otherwise noted, imaging was undertaken with a Deltavision RT Deconvolution microscope (Applied Precision) with an Olympus IX70 microscopy system (Olympus U-RFL-T and IX-HLSH100 lamps, and Olympus UPlanApo 100x/1.35 Oil Iris Lens), with Softworx 3.5.1 software. Cells were fixed for 30 min in 4% (v/v) para-formaldehyde before analysis by fluorescence microscopy. Fluorescence images are shown using a linear lookup table.

Deconvolution (iterative constrained deconvolution) was carried out using Softworx (3.5.1).

### Super resolution radial fluctuation (SRRF) imaging

SRRF imaging was carried out on a Nikon N-STORM microscope with a 100x objective (Plan-APOCHROMAT 100x/1.49 Oil, Nikon) and additional 1.5x magnification to collect fluorescence onto an EMCCD camera (iXon Ultra 897, Andor). Samples were prepared as follows. 10 µl of exponentially grown, fixed *S. aureus* SH1000 JGL232 (*plsY-gfp*) were resuspended in PBS and placed onto #1.5 thickness clean coverslips coated in poly-L-lysine and left for 20 min to settle. The coverslip was then washed once in milli-Q water and mounted in 100 mM mercaptoethylamine before imaging. For each SRRF image, 500 frames were acquired with a 488 nm laser operating at 100% power and 10 ms exposure time. The resultant time series were processed with the NanoJ-SRRF software package in Fiji.

### Whole culture measurements of GFP expression

IPTG controlled *plsY-gfp* expression from a plasmid pWhiteWalker10 was carried out by diluting overnight cultures to an OD_600_ = 0.05 followed by incubation at 37 °C, 250 rpm, until an OD_600_ ~ 1. 50 or 500 μM IPTG were added to induce the expression of *plsY-gfp*. Samples were taken prior to and 1, 2 and 3 h post induction. Fluorescence of samples was measured using a Tecan plate reader with 100 μl cells adjusted to an OD_600_ = 5 that were washed and resuspended in PBS, followed by exposure for 1 sec at 485 nm and emission at 535 nm.

### Protein-protein interaction studies

Fresh transformants of RN4220 carrying pWhiteWalker plasmids were used for FRET experiments. Overnight cultures were diluted to OD_600_ = 0.025 in 50 ml BHI supplemented with erythromycin (5 µg ml^−1^) and lincomycin (25 µg ml^−1^) and grown at 37 °C, 250 rpm, for 2.5 h to an OD_600_ ≈ 0.4. Cultures were diluted again to OD_600_ = 0.025 in 50 ml BHI supplemented with erythromycin (5 µg ml^−1^), erythromycin (25 µg ml^−1^) and 0.5 mM IPTG followed by incubation at 37 °C, 250 rpm, for 2 h to an OD_600_ ≈ 0.4. 1 ml samples were harvested by centrifugation at 13,000 rpm for 3 min and samples were washed with 1 ml PBS, fixed and prepared on poly-lysine slides.

Image acquisition was carried out using a Nikon Ti Eclipse inverted microscope and NIS elements software under a 100x oil lens in the FITC channel. The following settings were used for imaging:

$$\begin{array}{ll}{\rm{Format}}\mbox{--}{\rm{Binning}}\,2\times 2 & {\rm{Exposure}}\mbox{--}{\rm{500}}\,{\rm{ms}}\\ {\rm{Readout}}\,{\rm{mode}}\mbox{--}{\rm{Global}}\,{\rm{Shutter}} & {\rm{Readout}}\,{\rm{rate}}\mbox{--}{\rm{560}}\,{\rm{MHz}}\\ {\rm{Dynamic}}\,{\rm{Range}}\mbox{--}{\rm{12}}\,{\rm{bit}}\,\& \,{\rm{Gain}}\,1 & {\rm{Sensor}}\,{\rm{mode}}\mbox{--}{\rm{Normal}}\end{array}$$

Images were taken continuously over 6 min at 488 nm and fluorescence intensity over time was used to determine the photobleaching decay rate in arbitrary units/frame. These values were used to calculate the FRET efficiency using the following formula:

1 $${\rm{E}}=1-{{\rm{\tau }}}_{{\rm{PB}}}/{\rm{\tau }}{\mbox{'}}_{{\rm{PB}}}$$

where τ_PB_ is the time constant of PlsY-GFP in the absence of an acceptor and τ’_PB_ is the time constant of PlsY-GFP in presence of the investigated fusion. In other words, expression of *plsY-gfp* from pWhiteWalker10 was used for the determination of τ_PB_.

### Quantitative Image Analysis

#### Mander’s Coefficient

Both Mander’s coefficient and Pearson’s coefficient analyses were tested, leading to the same conclusions. However, Mander’s coefficient has several theoretical advantages for our data^64^.

Samples were prepared and imaged the same way as described before and three image fields with comparable amounts of cells per strain were taken. Images were deconvolved and both convolved and deconvolved images were analysed. Channels were separated and the background of fluorescence images was subtracted (Rolling Ball radius: 50 pixels). The threshold of the FITC channel image was auto adjusted and a selection of pixels within the threshold was created. Manders (M1) coefficient was determined using the Coloc2 Plugin in Fiji ImageJ using the threshold selection for both images. This procedure was repeated for all three replicates.

#### Coefficient of variation

Fluorescence images were converted to polar coordinates using the polar transformer plug-in for Fiji-ImageJ (https://imagej.nih.gov/ij/plugins/polar-transformer.html) where the y-value represents the angle and the x-value stands for the distance from the centre of the image. An intensity profile of this image was then created and plotted. The intensity values were used to calculate the standard deviation as an indicator for heterogeneity. The standard deviation is both influenced by the variation of fluorescence as well as the intensity. To remove the latter effect, the coefficient of variation (CV) was calculated as follows:

2 $$CV=\frac{\sigma }{100\,\mu }$$

where σ and µ are the standard deviation and mean of the distribution of fluorescence by angle (Fig. S2) and 100 is an arbitrary scaling factor.

A high CV indicates a more heterogeneous distribution of fluorescence signal, while low values indicate a more homogeneous distribution. Since cytoplasmic signals affect the CV the measurements were further optimised by removing the cytoplasm. This was performed by deleting approximately 20% of the cell volume from the cell centre. However, this procedure was not carried out for deconvolved images since these had very low cytoplasmic signals. CV measurements were made using *n* = 20 cells per group unless otherwise stated.

### Statistics

Standard deviations and student t-tests were calculated on at least three experimental replicates using GraphPad Prism 6.05 (GraphPad Software Inc., La Jolla, California).

## Electronic supplementary material

Weihs et al Supplementary Information
