# Supplementary material for: Heterogeneous localisation of membrane proteins in Staphylococcus aureus
Source: Sci Rep. 2018 Feb 26;8:3657. doi: 10.1038/s41598-018-21750-x (PMC5826919; doi:10.1038/s41598-018-21750-x)
Supplement: Supplementary file 1 — Weihs et al Supplementary Information [file 41598_2018_21750_MOESM1_ESM.docx]

**Title**

Heterogeneous localisation of membrane proteins in *Staphylococcus aureus*

**Authors and affiliations**

Felix Weihs^1^, Katarzyna Wacnik^1^, Robert D. Turner^1^, Siân Culley^2^, Ricardo Henriques^2^ & Simon J. Foster^1*^

^1^ The Krebs Institute. Department of Molecular Biology and Microbiology, University of

Sheffield, Firth Court, Western Bank, Sheffield S10 2TN, UK.

^2^ Quantitative Imaging and Nanobiophysics Group, MRC Laboratory for Molecular Cell Biology and Department of Cell and Developmental Biology, University College London, Gower Street, London, WC1E 6BT, UK.

* Corresponding author: s.foster@sheffield.ac.uk

Contact Information:

Professor Simon J. Foster, Department of Molecular Biology and Biotechnology, University of Sheffield, Firth Court, Western Bank, Sheffield, S10 2TN, UK

Email: s.foster@sheffield.ac.uk

**Supplementary Information**

**Supplementary Figure S1** The localisation of PlsY

**Supplementary Figure S2** Quantitative image analyses of membrane protein distributions

**Supplementary Figure S3** Bioinformatic analysis of phospholipid synthesis enzymes

**Supplementary Figure S4.** Western blots confirm the expression of single-copy eYFP/GFP fusions and FRET construct mCherry fusions

**Supplementary Figure S5** The three-dimensional distribution of PlsY

**Supplementary Figure S6** Inhibition of squalene production by zaragozic acid does not affect the localisation of PlsY

**Supplementary Figure S7** Fatty-acid synthesis inhibition and membrane potential inhibition do not clearly affect PlsY-localisation

**Supplementary Figure S8** SDS treatment causes the disruption of the localisation pattern of PlsY

**Supplementary Figure S9** *tarO* cells exhibit increased cell sizes

**Supplementary Figure S10** FtsZ inhibition disrupts the localisation pattern of PlsY

**Supplementary Table 1** Bacterial strains and plasmids used in this study

**Supplementary Table 2** Oligonucleotides used in this study

**Methods** - Construction of bacterial strains/plasmids and Western blotting

**Supplementary References**


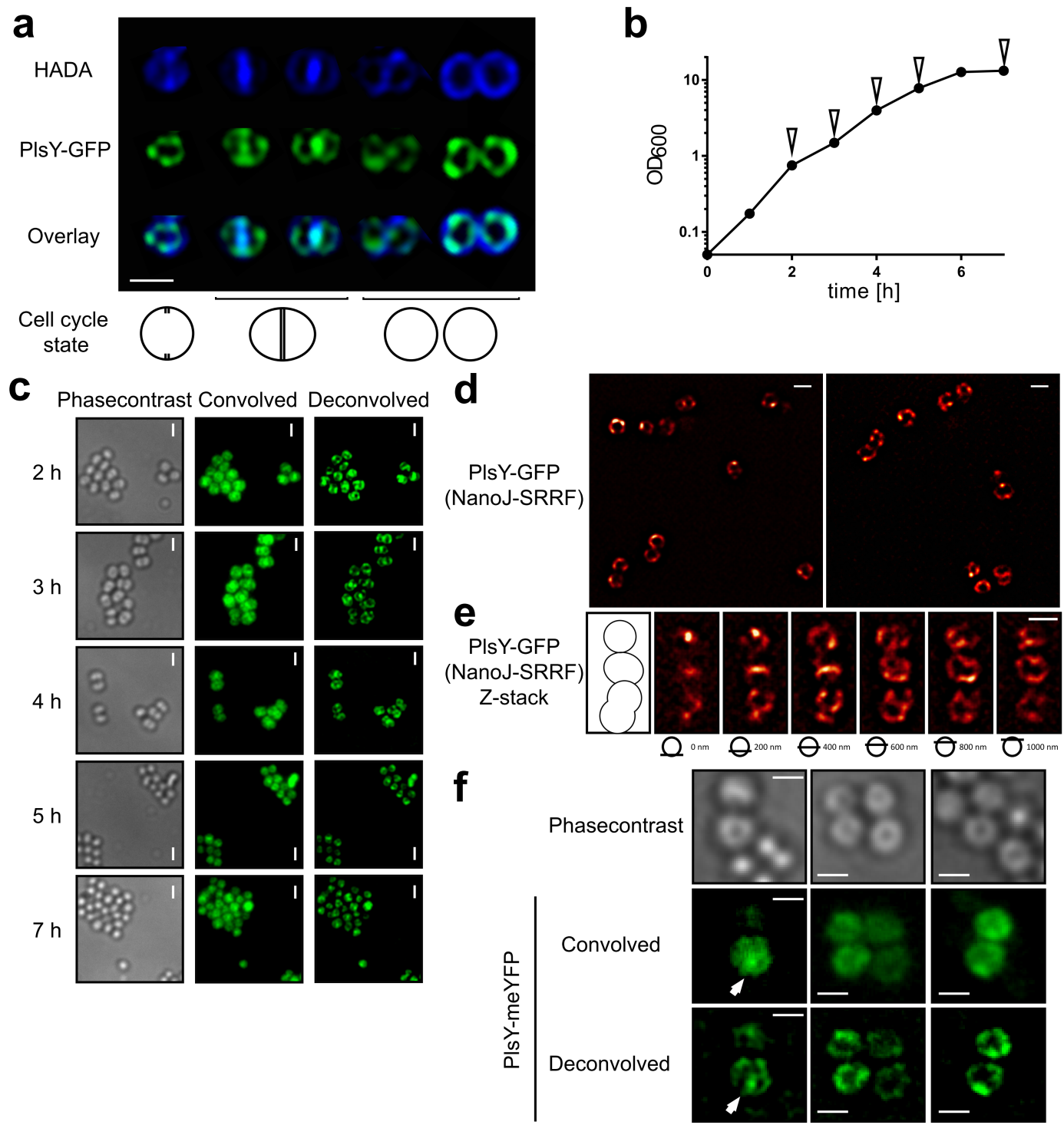


**Figure S1. The localisation of PlsY**

**a**, Cell-cycle dependent localisation of PlsY-GFP. Deconvolved fluorescence images of selected cells representing different cell cycle states of *S. aureus* SH1000 JGL232 (*plsY-gfp*) labelled with HADA for 5 min. **b, c**, Growth-phase dependent localisation of PlsY-GFP in *S. aureus* SH1000 JGL232 (*plsY-gfp*). The growth curve using optical density measurements at 600 nm indicates when samples were taken. Phase contrast and fluorescence images (convolved and deconvolved) of *S. aureus* SH1000 JGL232 expressing *plsY-gfp* at early-exponential (2 and 3 h post inoculation), late-exponential (4 and 5 h) and stationary (7 h) growth phase. **d**, Nano-J SRRF imaging of exponentially growing *S. aureus* SH1000 JGL232 (*plsY-gfp)* cells. **e**, Nano-J SRRF imaging of exponentially growing *S. aureus* SH1000 JGL232 (*plsY-gfp*) cells in a Z-stack series. **f**, Phase contrast and fluorescence images (convolved and deconvolved) of *S. aureus* SH1000 FW10 (*plsY-meyfp*). All scale bars represent 1 µm.

**
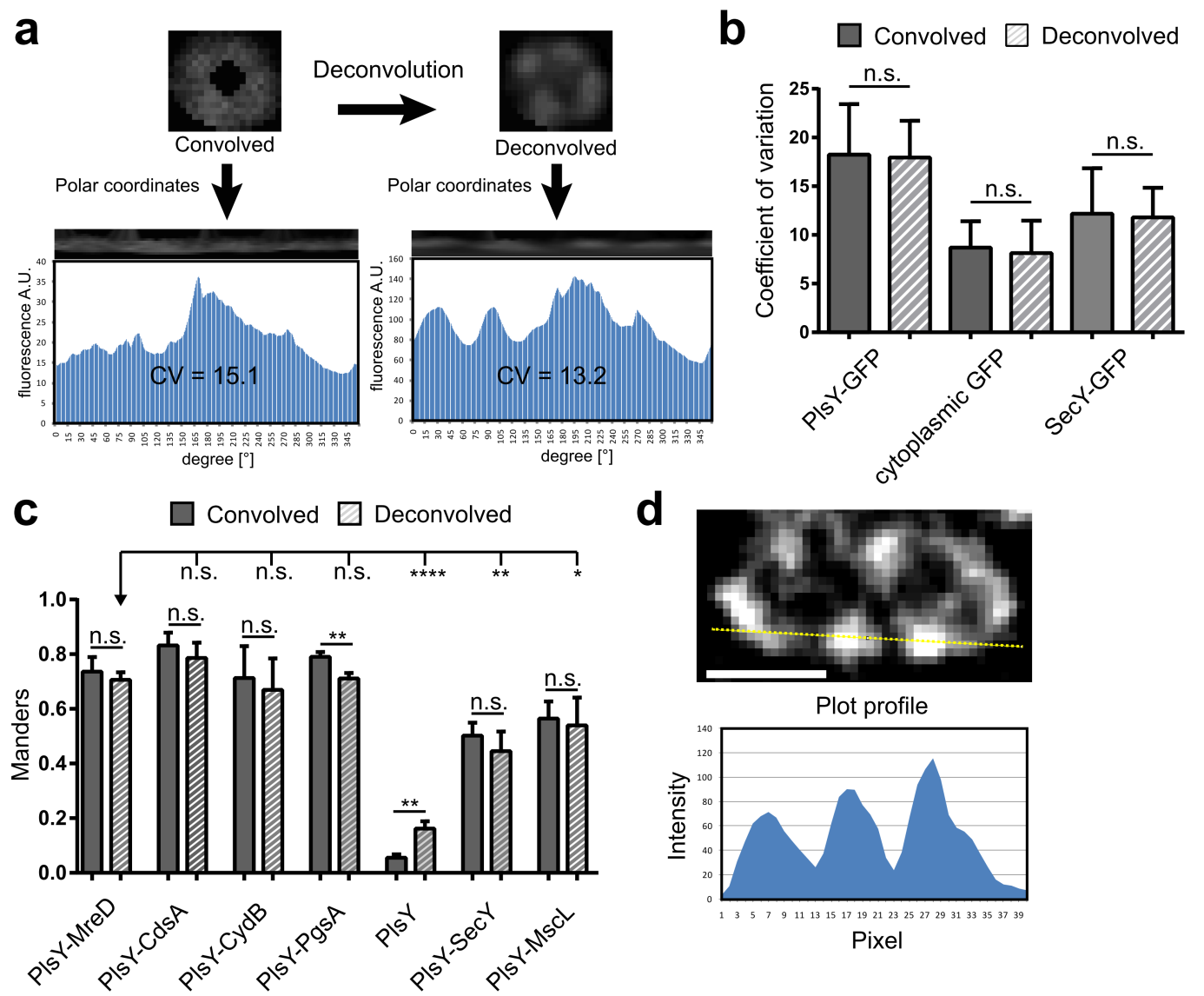
**

**Figure S2. Quantitative image analyses of membrane protein distributions**

**a**, Example of Coefficient of Variation (CV) calculation for a cell expressing *plsY-gfp* (JGL232). The image was converted into polar coordinates and the CV was calculated based on the distribution of fluorescence signal within a plot profile. This procedure was repeated with the same deconvolved image to analyse differences introduced by deconvolution. To reduce the impact of apparent cytoplasmic signals in convolved images, approximately 20 % of the total cell volume from the middle of the cell were deleted **b**, Demonstration that deconvolution of images does not affect the CV significantly using 10 cells of *S. aureus* SH1000 expressing *plsY-gfp* (JGL232), expression of cytoplasmic *mCherry-gfp* (FW13) and *secY-gfp* (JGL231). Open bars convolved. Striped bars deconvolved. **c**, Manders analysis of convolved (open bars) and deconvolved (striped bars) images of cells (strains FW14-FW20) expressing *plsY-gfp* and a gene of interest fused to *mCherry*. The analysis was carried out three times of whole image fields and p-values were calculated using a two-tailed, unpaired student t-test. **P < 0.01. All significance values were calculated using a two-tailed unpaired student t-test. **d**, Example for the CV-factor calculation of *E. coli* rod-shaped cells using the fluorescence signal profile of the longitudinal axis. Scale bar represents 1 µm.

**
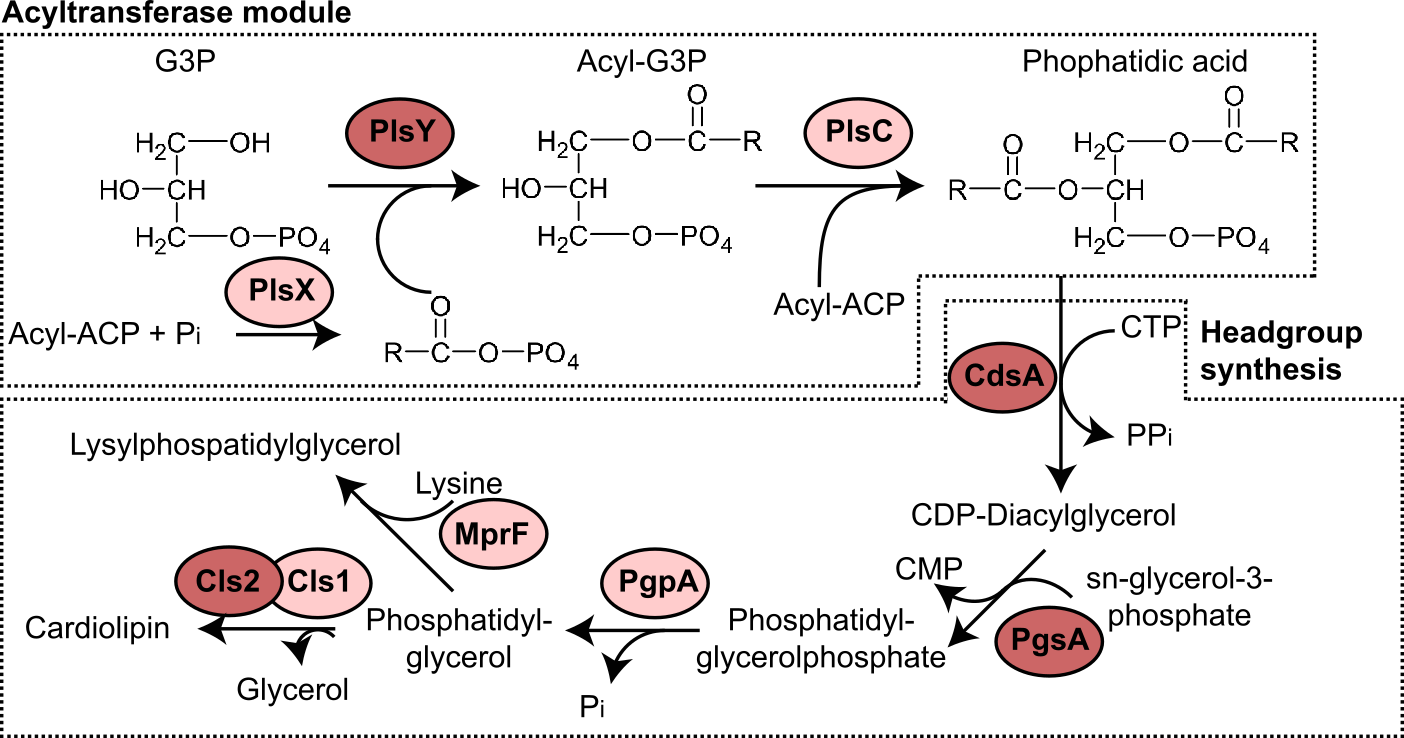
**

**Figure S3. Overview of phospholipid synthesis in *S. aureus***

Phospholipid biosynthesis is catalysed from glycerol-3-phosphate firstly by the acyltransferase module which incorporates Acyl-ACP into G3P using the PlsX/PlsY/PlsC system. Then phospholipid head group synthesis occurs. Phosphatidic acid gets cytidinylated to CDP-diacylglycerol by CdsA which is then further processed to phosphatidylserine and eventually phosphatidylethanolamine by PssA and Psd. Alternatively, CDP-diacylglycerol is converted to phosphatidylglycerolphosphate by PgsA, followed by dephosphorylation to phosphatidylglycerol catalysed by the enzyme PgpA. Phosphatidylglycerol is then either converted to cardiolipin via Cls1/2 or lysinylated by MprF to lysylphophatidylglycerol. Figure is adapted from Parsons and Rock, 2013^1^. The enzyme components involved are encircled and those analysed in this study have the darker shading.


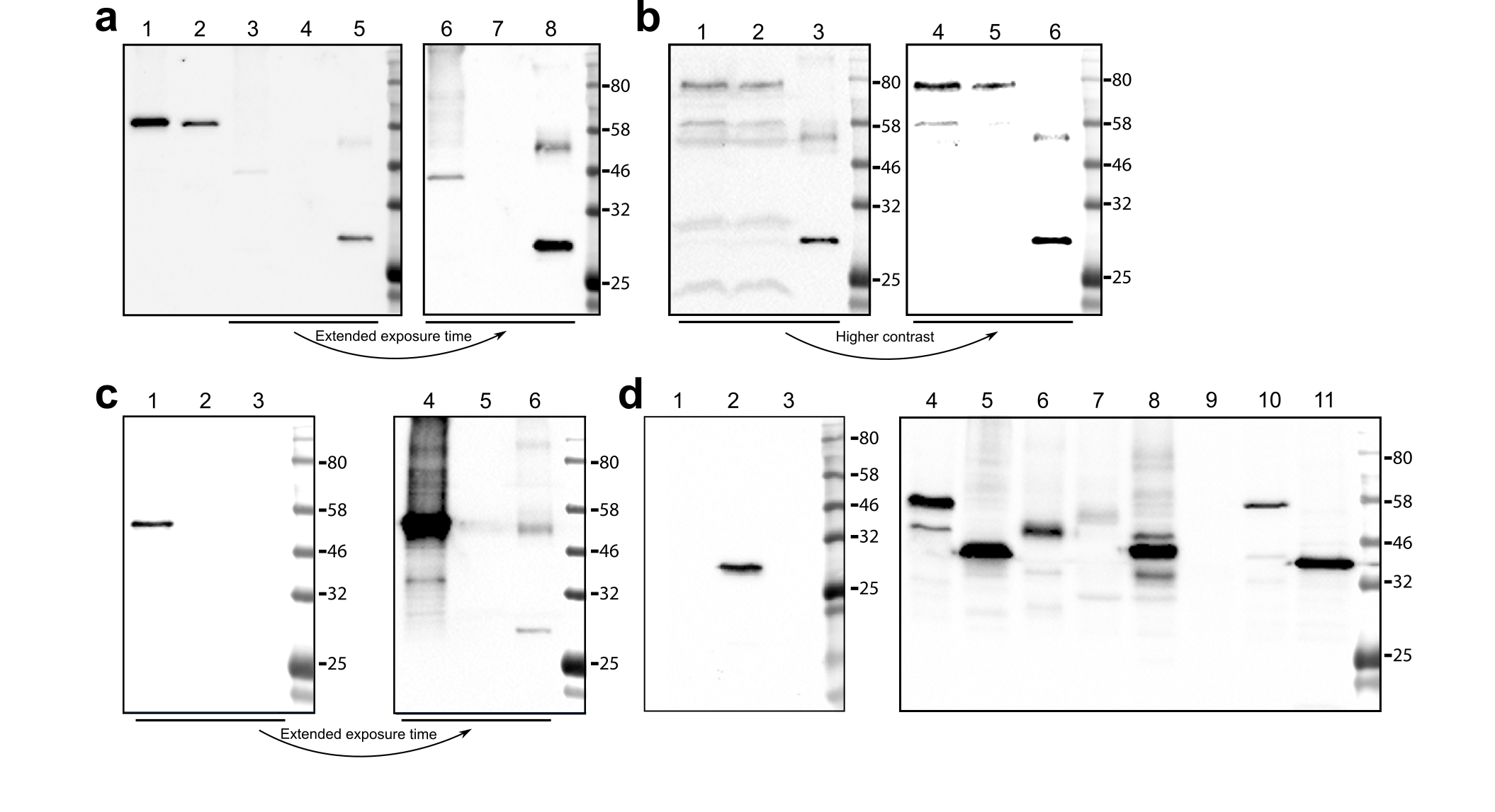


**Figure S4. Western blots confirm the expression of single-copy eYFP/GFP fusions and FRET construct mCherry fusions.**

**a-c**, Western blot analysis of membrane fractions isolated from *S. aureus* strains using anti-GFP antibodies. Purified GFP was used as a positive control whereas membrane fractions collected from SH1000 were used as a negative control. **a**, SH1000 JGL231 (*secY-gfp)* (lane 1), FW8 (*floT-eyfp)* (lane 2), FW2 (*pgsA-eyfp)* (lane 3), SH1000 (lane 4) and 50 ng of purified GFP (lane 5). A prolonged exposure time was used for FW2 (*pgsA-eyfp)*, SH1000 and 50 ng of purified GFP (lanes 6-8) to accentuate the band corresponding to PgsA-eYFP at ~45 kDa (lane 6). Sizes of a protein ladder are shown in kDa. Predicted molecular weights of GFP/eYFP fusions are as follows: SecY-GFP (74 kDa); FloT-eYFP (61 kDa) and PgsA-eYFP (47 kDa). **b**, FW1 (*plsY-eyfp)* (lane 1), SH1000 (lane 2) and 50 ng of purified GFP (lane 3). A higher contrast version of the image is shown on the right panel (lanes 4-6) to accentuate the band corresponding to PlsY-eYFP at ~55 kDa (lane 4). Sizes of a protein ladder are shown in kDa. The predicted molecular weight for PlsY-eYFP is 49 kDa. **c**, FW5 (*∆geh::P_cls2_:cls2~eyfp*) (lane 1), SH1000 (lane 2) and 50 ng of purified GFP (lane 3). A prolonged exposure time was used to show the band corresponding to purified GFP (lane 6) (lanes 6-8). Sizes of a protein ladder are shown in kDa. The predicted molecular weight for Cls2-eYFP is 83 kDa. **d**, Western blots using anti-mCherry antibodies of IPTG-induced and lysed cells of *S. aureus*. Whole cell lysates of SH1000 (lane 1), SH1000 expressing *mCherry* from plasmid pMV1580-mCherry (lane 2) and SH1000 expressing *gfp* from plasmid pMV1580-gfp (lane 3) were used as controls. FW13 (*gfp-mCherry* (tandem)) (lane 4), FW14 (*mreD-mCherry + plsY-gfp*) (lane 5), FW15 (*cdsA-mCherry + plsY-gfp*) (lane 6), FW16 (*cydB-mCherry + plsY-gfp*) (lane 7), FW17 (*pgsA-mCherry + plsY-gfp*) (lane 8), FW18 (*plsY-gfp*) (lane 9), FW19 (*secY-mCherry + plsY-gfp*) (lane 10) and FW20 (*mscL-mCherry + plsY-gfp*) (lane 11). Sizes of a protein ladder are shown in kDa. Predicted molecular weights of mCherry fusions are as follows: GFP-mCherry (tandem) (54 kDa); MreD-mCherry (59 kDa); CdsA-mCherry (46 kDa), CydB-mCherry (66 kDa), PgsA-mCherry (48 kDa), SecY-mCherry (74 kDa), MscL-mCherry (41 kDa). Migration of some of the fusion proteins such as SecY-mCherry, SecY-GFP, PlsY-eYFP, Cls2-eYFP and MreD-mCherry does not correlate with their respective formula molecular weights which could be due to detergent binding induced “gel shifting”^17^.


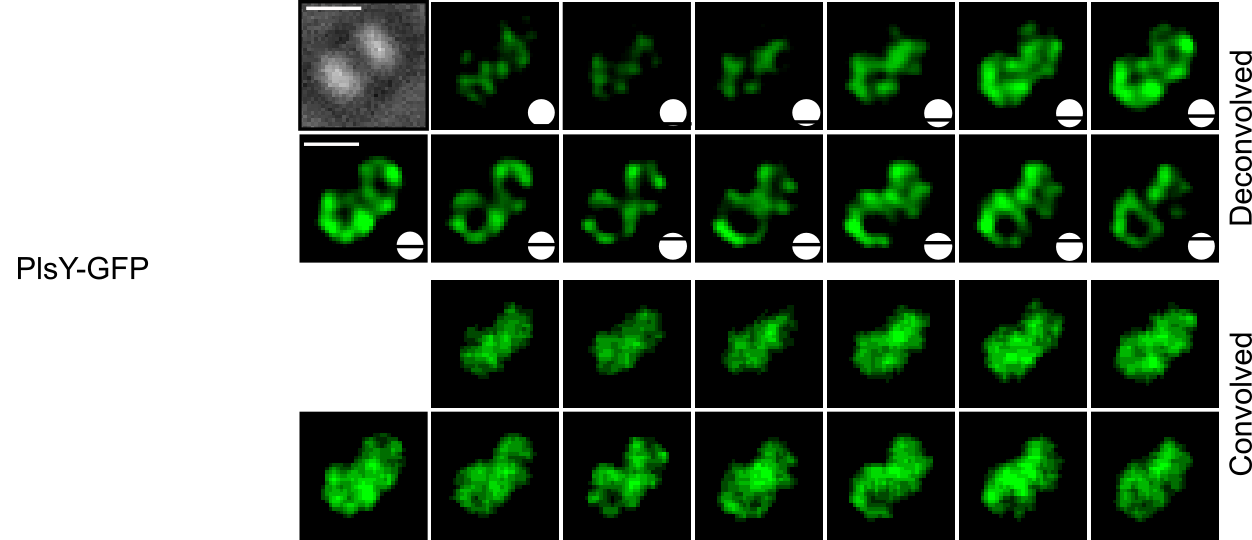


**Figure S5. The three-dimensional distribution of PlsY**

Z-stack image series of selected cells of *S. aureus* SH1000 JGL232 (*plsY-gfp*) (100 nm steps indicated by the sphere within or above the fluorescence images) of fluorescence images (convolved and deconvolved). Scale bars represent 1 µm.


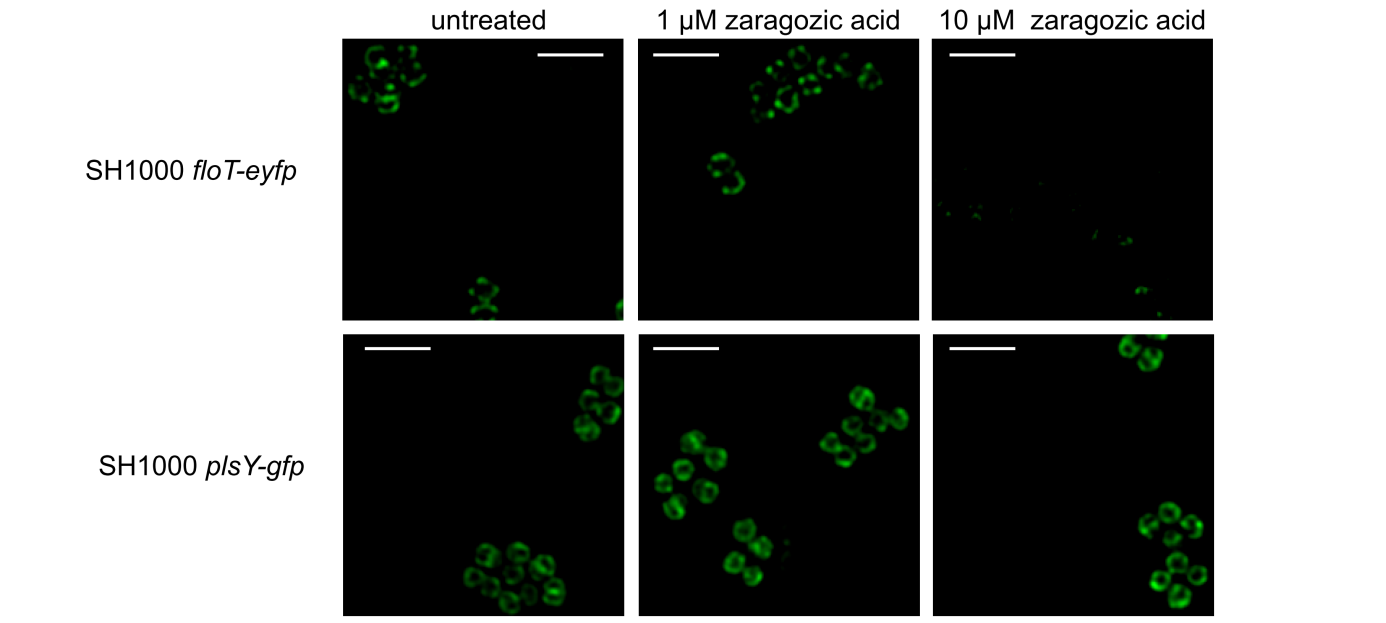


**Figure S6. Inhibition of squalene production by zaragozic acid does not affect the localisation of PlsY**

Fluorescence images (deconvolved) of *S. aureus* SH1000 FW8 (*floT-eyfp*) and SH1000 JGL232 (*plsY-gfp)* after 2 h treatment with 1 or 10 µM zaragozic acid. Scale bars represent 3 µm.


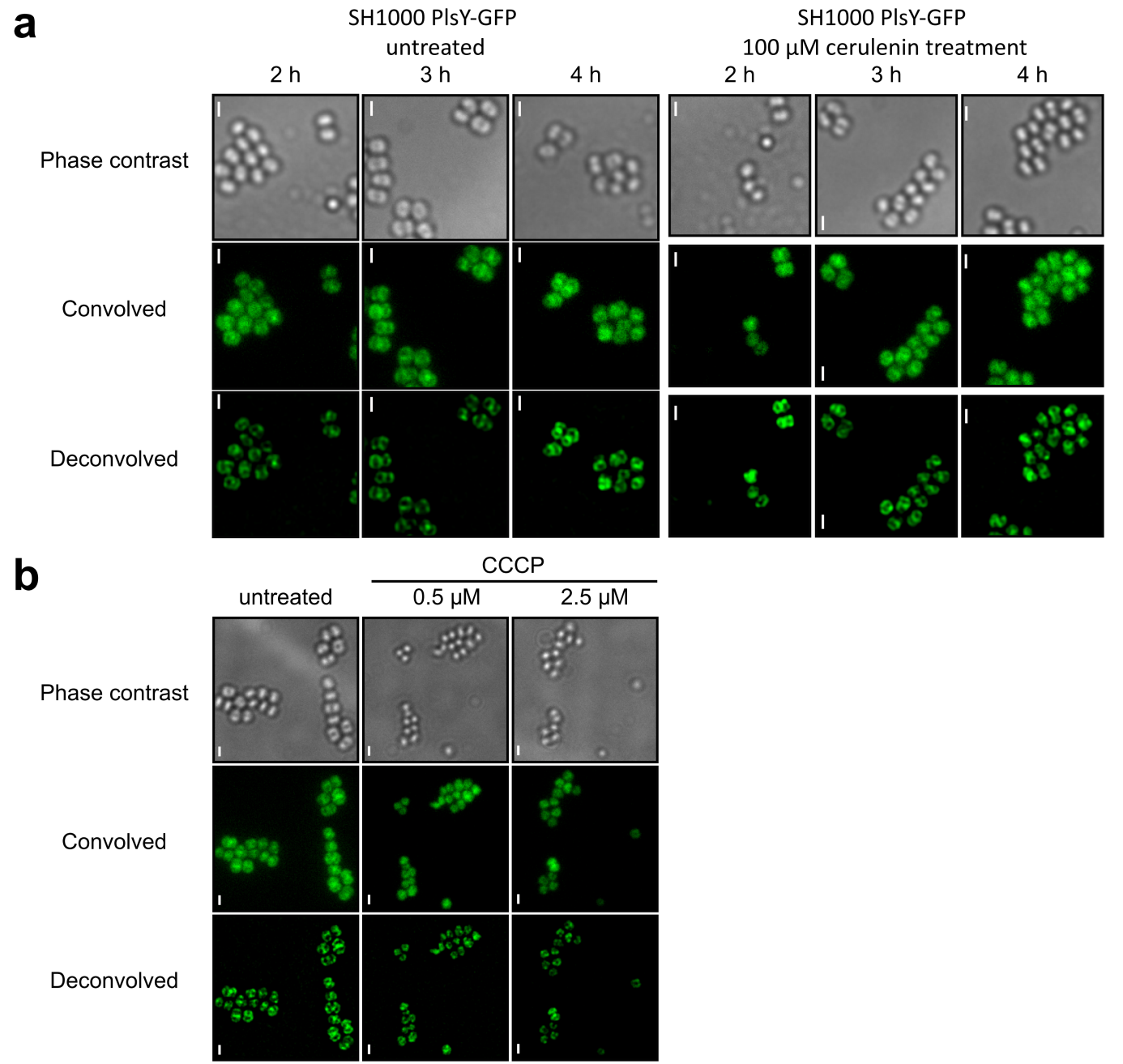


**Figure S7. Fatty-acid synthesis inhibition and membrane potential inhibition do not clearly affect PlsY-localisation**

**a**, Phase contrast and fluorescence images (convolved and deconvolved) of *S. aureus* SH1000 JGL232 (*plsY-gfp*) samples taken 2, 3 and 4 h after inoculation. **b**, Phase contrast and fluorescence images (convolved and deconvolved) of *S. aureus* SH1000 JGL232 (*plsY-gfp*) in the presence of different amounts of [carbonyl cyanide m-chlorophenyl hydrazone](https://en.wikipedia.org/wiki/Carbonyl_cyanide_m-chlorophenyl_hydrazone) (CCCP). All scale bars represent 1 µm.


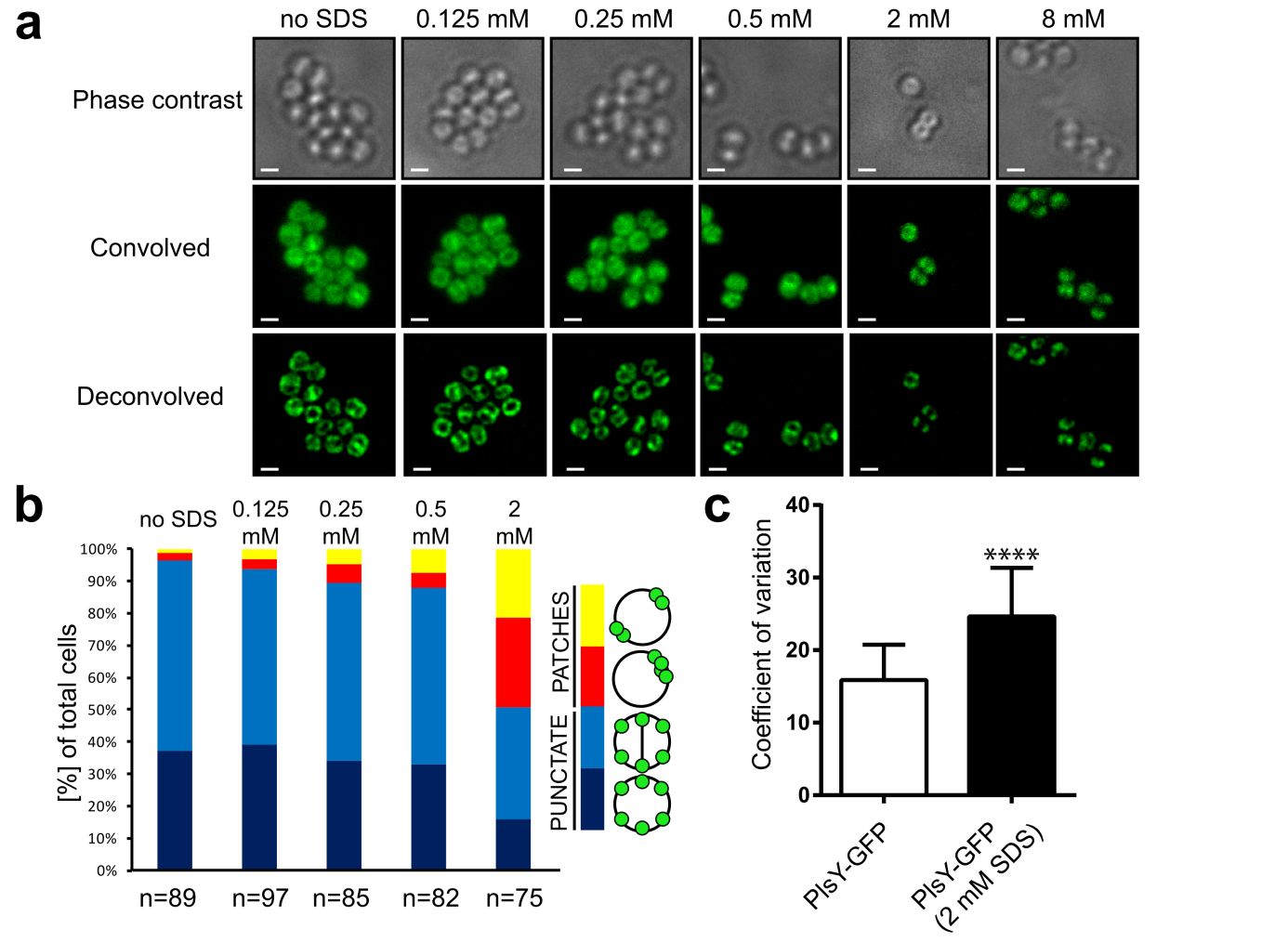


**Figure S8. SDS treatment causes the disruption of the localisation pattern of PlsY**

**a**, Phase contrast and fluorescence images (convolved and deconvolved) of *S. aureus* SH1000 JGL232 (*plsY-gfp*) samples treated with different SDS concentrations. Scale bars represent 1 µm. **b**, Cell counts of PlsY-GFP localisation categorised in 4 groups. Red and yellow bars indicate localisation of PlsY-GFP in one or two patches in the membrane. Blue bars indicate PlsY-GFP localisation in a punctate heterogeneous pattern and at the septum in dividing cells. N indicates number of cells counted for each group. **c**, CV-factor calculation of deconvolved images of JGL232 (*plsY-gfp)* untreated and treated with 2 mM SDS using 20 cells each. Statistical significance was calculated using a two-tailed unpaired student t-test. ****P < 0.0001.


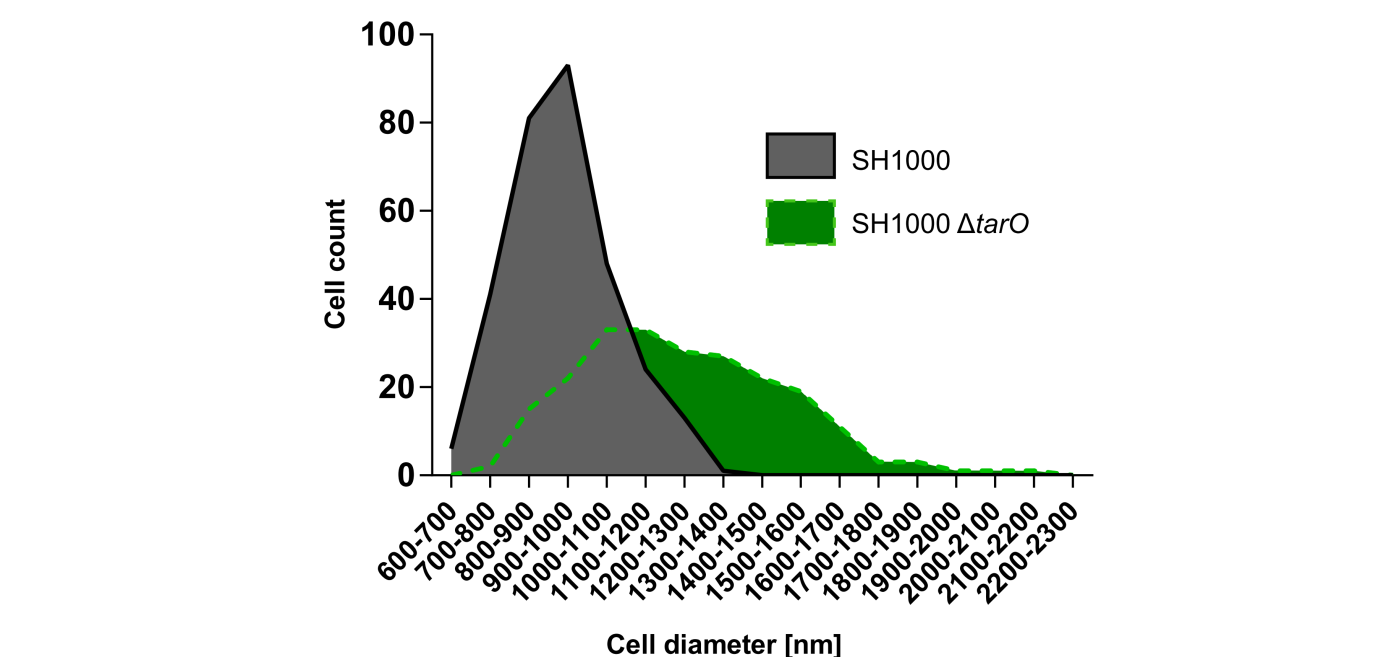


**Figure S9. *tarO* cells exhibit increased cell sizes**

Cell counts of *S. aureus* SH1000 and *S. aureus* SH1000 ∆*tarO* exponentially growing cells, categorised into their cell diameter.


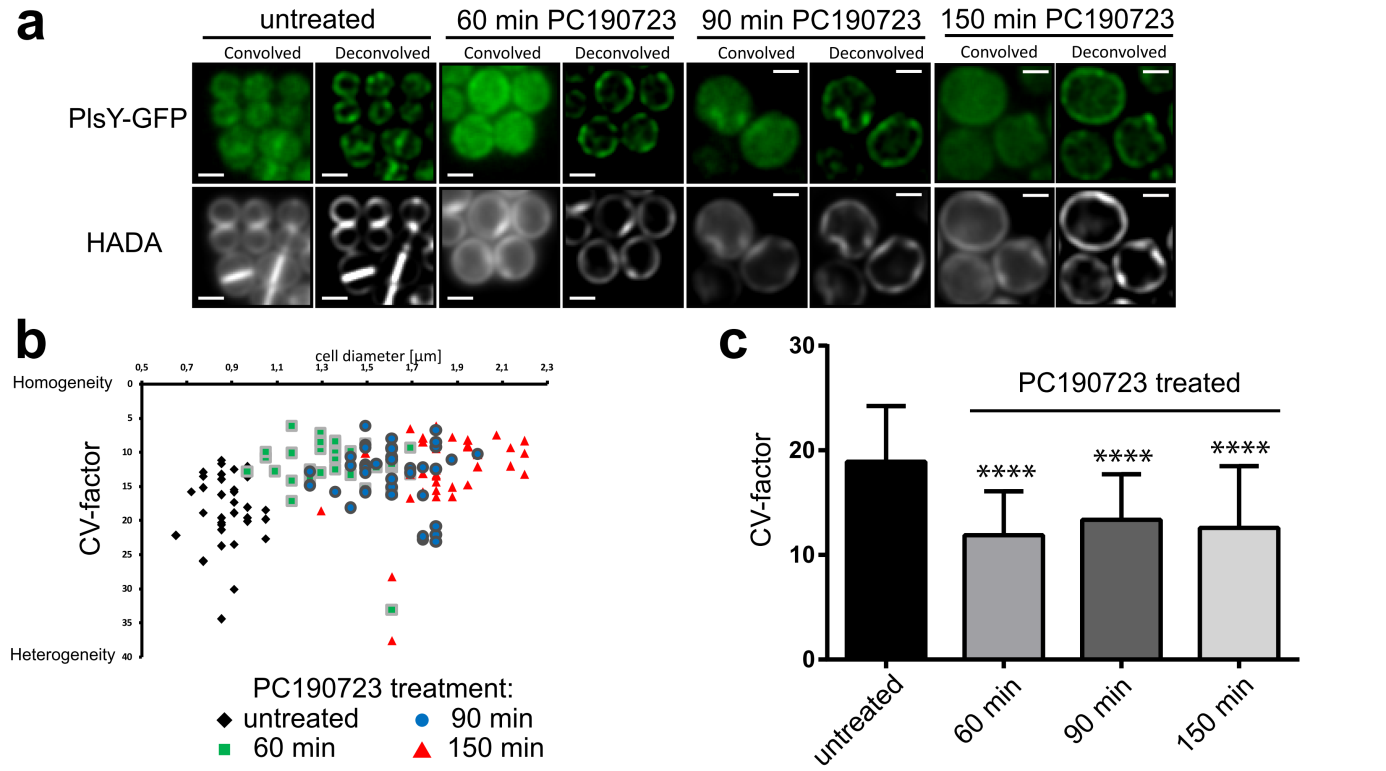


**Figure S10. Inhibition of FtsZ-polymerisation disrupts the localisation pattern of PlsY**

**a**, Fluorescence images (deconvolved) of *S. aureus* SH1000 JGL232 (*plsY-gfp)* samples treated or untreated with PC190723. Samples were taken before or 60, 90 and 150 min after addition of PC190723 with HADA labelling for the final 30 min. Scale bars represent 1 µm. **b**, Fluorescence images (deconvolved) of JGL232 (*plsY-gfp)* cells untreated (diamonds) or treated with PC190723 for 60 min (green squares), 90 min (blue circles) or 150 min (red triangles) were measured in diameter (X-axis) and CV-factor (Y-axis) and plotted. Each data point represents a single cell. **c**, CV-factor calculation of deconvolved images of JGL232 (*plsY-gfp)* untreated and treated with PC190723 for 60, 90 or 150 min. 40 cells were measured for each group and significance values against the untreated group were calculated using a two-tailed unpaired student t-test. ****P < 0.0001.

**Supplementary Table 1. Bacterial strains and plasmids used in this study**

| **Strain** | **Relevant features** | **Reference** |
| --- | --- | --- |
| ***Escherichia coli* strains** |  |  |
| NEB5α | fhuA2 Δ(argF^-^lacZ)U169 phoA glnV44 Φ80 Δ(lacZ)M15 gyrA96 recA1 relA1 endA1 thi-1 hsdR17 | New England Biolabs |
| DC10B | *mcrA* Δ*mrr-hsdRMS-mcrBC) φ80lacZ*Δ*M15* Δ*lacX74 recA1 araD139* Δ*(ara-leu)7697 galU galK rpsL endA1 nupG* Δ*dcm* | [2](#_ENREF_1) |
| C43(DE3) | F^-^ ompT hsdSB (rB^-^ mB^-^) gal dcm (DE3) | [3](#_ENREF_2) |
| C43(DE3) *mreD-eyfp* | pWALDO-*mreD-eyfp*; IPTG-inducible expression of *mreD*~*eyfp* ; Kan^R^ (*E. coli*) | This study |
|  |  |  |
| **Plasmids** |  |  |
| pMUTIN-*gfp*+ | GFP+ containing vector; Amp^R^ (*E. coli*), Ery^R^,Lin^R^ (*S. aureus*) | [4](#_ENREF_3) |
| pAISH1 | Similar to pMUTIN-*gfp*+ but with Tet^R^ (*S. aureus*), Amp^R^ (*E. coli*) | [5](#_ENREF_4) |
| pAISH-*plsY-gfp* | Non-replicating in *S. aureus*, integrates into the native locus of *plsY*, thereby tags *plsY* with *gfp* and places the original *plsY* under the IPTG-inducible promoter Pspac. Tet^R^ (*S. aureus*) Amp^R^ (*E. coli*) | This study |
| pMUTIN-*pgsA-eyfp* | Non-replicating in *S. aureus*, integrates into the native locus of *pgsA*, thereby tags *pgsA* with e*yfp* and places the original *pgsA* under the IPTG-inducible promoter Pspac. Ery^R^, Lin^R^ (*S. aureus*) Amp^R^ (*E. coli*) | This study |
| pMUTIN-*floT-eyfp* | Non-replicating in *S. aureus*, integrates into the native locus of *floT*, thereby tags *floT* with e*yfp* and places the original *floT* under the IPTG-inducible promoter Pspac. Ery^R^, Lin^R^ (*S. aureus*) Amp^R^ (*E. coli*) | This study |
| pWALDO-*gfp-*E | Overexpression vector, IPTG-inducible expression of an insert tagged to *gfp* and under the control of a T7 RNA polymerase promoter, expresses *lacI*; Kan^R^ (*E. coli*) | [6](#_ENREF_5) |
| pWALDO-*mreD-eyfp* | T7 RNA polymerase driven IPTG-inducible expression of C-terminal tagged *mreD* with *eyfp*; Kan^R^ (*E. coli*) | This study |
| pWhiteWalker2 | IPTG-inducible expression of *gfp* and *mCherry* fused in tandem; Ery^R^, Lin^R^ (*S. aureus*) Amp^R^ (*E. coli*) | [7](#_ENREF_6) |
| pWhiteWalker3 | IPTG-inducible expression of *plsY*~*gfp* and *mreD*~*mCherry*; Ery^R^, Lin^R^ (*S. aureus*) Amp^R^ (*E. coli*) | [7](#_ENREF_6) |
| pWhiteWalker4 | IPTG-inducible expression of *plsY*~*gfp* and *cdsA*~*mCherry*; Ery^R^, Lin^R^ (*S. aureus*) Amp^R^ (*E. coli*) | [7](#_ENREF_6) |
| pWhiteWalker7 | IPTG-inducible expression of *plsY*~*gfp* and *cydB*~*mCherry*; Ery^R^, Lin^R^ (*S. aureus*) Amp^R^ (*E. coli*) | This study |
| pWhiteWalker8 | IPTG-inducible expression of *plsY*~*gfp* and *pgsA*~*mCherry*; Ery^R^, Lin^R^ (*S. aureus*) Amp^R^ (*E. coli*) | This study |
| pWhiteWalker10 | IPTG-inducible expression of *plsY*~*gfp*; Ery^R^, Lin^R^ (*S. aureus*) Amp^R^ (*E. coli*) | [7](#_ENREF_6) |
| pWhiteWalker12 | IPTG-inducible expression of *plsY*~*gfp* and *secY*~*mCherry*; Ery^R^, Lin^R^ (*S. aureus*) Amp^R^ (*E. coli*) | [7](#_ENREF_6) |
| pWhiteWalker13 | IPTG-inducible expression of *plsY*~*gfp* and *mscL*~*mCherry*; Ery^R^, Lin^R^ (*S. aureus*) Amp^R^ (*E. coli*) | [7](#_ENREF_6) |
| pKASBAR | Hybrid vector of pCL84 and pUC18 for integration into *S. aureus* lipase gene (*geh*) encoding the *attP* integration site of L54a phage; Amp^R^ (*E. coli*), Tet^R^ (*S. aureus*) | [8](#_ENREF_7) |
| pKASBAR-*cls2-eyfp* | Non-replicating in *S.aureus*, integrates into lipase gene (*geh*), expression of *cls2~eyfp* under the putative promoter of *cls2*, Tet^R^ (*S.aureus*) Amp^R^ (*E.coli*) | This study |
| pKASBAR-*ezrA-eyfp* | pKASBAR-Kan^R^; expression of *ezrA*~*eyfp* under the putative promoter of *ezrA*; Amp^R^ (*E. coli*), Kan^R^ (*S. aureus*) | [9](#_ENREF_8) |
| pKASBAR-*ezrA-meyfp* | pKASBAR-Kan^R^; expression of *ezrA*~*meyfp* under the putative promoter of *ezrA*; Amp^R^ (*E. coli*), Kan^R^ (*S. aureus*) | [9](#_ENREF_8) |
| pMV158-*gfp* | Expression of *gfp*; Tet^R^ (*S. aureus*) | 18 |
| pMV158-*mCherry* | Expression of *mCherry*; Tet^R^ (*S. aureus*) | Tomasz Prajsnar |
|  |  |  |
|  |  |  |
| ***Staphylococcus aureus* strains** |  |  |
| SH1000 | 8325-4 *rsbU*^+^ | [10](#_ENREF_9) |
| JGL231 | SH1000; P_secY_:*secY*~*gfp*+ P_spac_:*secY*; Ery^R^, Lin^R^ | [7](#_ENREF_6) |
| JGL232 | SH1000; P_plsY_:*plsY*~*gfp*+ P_spac_:*plsY*; Ery^R^, Lin^R^ | [7](#_ENREF_6) |
| FW1 | SH1000; P_plsY_:*plsY*~*eyfp*+ P_spac_:*plsY*; Ery^R^, Lin^R^ | This study |
| FW2 | SH1000; P_pgsA_:*pgsA*~*eyfp*+ P_spac_:*pgsA*; Ery^R^, Lin^R^ | This study |
| FW5 | RN4220; ∆*cls2::Tn*; integrated pKASBAR-*cls2-eyfp* resulting in: ∆*geh::*P_cls2_:*cls2*~*eyfp;* Ery^R^, Lin^R^, Tet^R^ | This study |
| FW6 | SH1000; pWhiteWalker10 (IPTG-inducible expression of *plsY-gfp*); Ery^R^, Lin^R^ | This study |
| FW8 | SH1000; P_floT_:*floT*~*eyfp*+ P_spac_:*floT;* Ery^R^, Lin^R^ | This study |
| FW9 | SH1000; P_plsY_:*plsY*~*gfp*+ P_spac_:*plsY*; Tet^R^ | This study |
| FW10 | SH1000; P_plsY_:*plsY*~*meyfp* P_spac_:*plsY*; Ery^R^, Lin^R^ | This study |
| FW13 | RN4220; pWhiteWalker2; Ery^R^, Lin^R^ | [7](#_ENREF_6) |
| FW14 | RN4220; pWhiteWalker3; Ery^R^, Lin^R^ | [7](#_ENREF_6) |
| FW15 | RN4220; pWhiteWalker4; Ery^R^, Lin^R^ | [7](#_ENREF_6) |
| FW16 | RN4220; pWhiteWalker7; Ery^R^, Lin^R^ | This study |
| FW17 | RN4220; pWhiteWalker8; Ery^R^, Lin^R^ | This study |
| FW18 | RN4220; pWhiteWalker10; Ery^R^, Lin^R^ | [7](#_ENREF_6) |
| FW19 | RN4220; pWhiteWalker12; Ery^R^, Lin^R^ | [7](#_ENREF_6) |
| FW20 | RN4220; pWhiteWalker13; Ery^R^, Lin^R^ | [7](#_ENREF_6) |
| FW21 | SH1000; ∆*cls1::cat*; ∆*cls2::tet*; P_plsY_:*plsY*~*gfp*+ P_spac_:*plsY*; Tet^R^, Ery^R^, Lin^R^, Cm^R^ | This study |
| FW22 | SH1000; ∆*mprF::ermB*; P_plsY_:*plsY*~*gfp*+ P_spac_:*plsY*; Tet^R^, Ery^R^, Lin^R^ | This study |
| FW23 | SH1000; ∆*tarO::ermB*; P_plsY_:*plsY*~*gfp*+ P_spac_:*plsY*; Tet^R^, Ery^R^, Lin^R^ | This study |
| SA113 Δ*tarO*+ pRB‑*tarO* | SA113 Δ*tarO::ermB* + pRB‑*tarO*; Ery^R^, Cm^R^ | 15 |
| SH1000 Δ*tarO* | SH1000 Δ*tarO::ermB*; Ery^R^ | This study |
| JE NE258 | JE2 *cls2::Tn*; from NARSA transposon library, transposon inserted at the beginning of *cls2*; Ery^R^ | 13 |

Ery^R^, erythromycin resistance; Lin^R^, lincomycin resistance; Tet^R^, tetracycline resistance, Cm^R^, chloramphenicol resistance; Kan^R^, kanamycin resistance; Amp^R^, ampicillin resistance.

**Supplementary Table 2. Oligonucleotide primers used in this study**

| Oligonucleotide name | Oligonucleotide sequence (5’- 3’) | Construction of : |
| --- | --- | --- |
| 5’FW09 | cctttttttgccccggcaaactaaattgtcatgatatttaaattg | Amplification of *cls2* and its upstream sequence containing its putative promoter for Gibson Assembly of pKASBAR-*cls2-eyfp* |
| 3’FW09 | gaacctgaacctgaggataagataggtgacaataattgtg | Amplification of *cls2* and its upstream sequence containing its putative promoter for Gibson Assembly of pKASBAR-*cls2-eyfp* |
| 5’FW10 | ctagagtcgagggtacatgtattcagtgattagtaagattttg | Amplification of *eyfp* for Gibson Assembly of pMUTIN-*plsC-eyfp* |
| 5’FW16 | ctagagtcgagggtacatgaatattccgaaccagattac | Amplification of *pgsA* for Gibson Assembly of pMUTIN-*pgsA-eyfp* |
| 3’FW16 | gaacctgatttttgtttaaaaacatctctacctttataaaag | Amplification of *pgsA* for Gibson Assembly of pMUTIN-*pgsA-eyfp* |
| 5’FW17 | tttaaacaaaatcaggttcaggttcaggtatg | Amplification of *eyfp* for Gibson Assembly of pMUTIN-*pgsA-eyfp* |
| 5’FW19 | ctagagtcgagggtacatgtttagtttaagttttatcgtaatag | Amplification of *floT* for Gibson Assembly of pMUTIN-*floT-eyfp* |
| 3’FW19 | gaacctgaatgttcaggtgactcatc | Amplification of *floT* for Gibson Assembly of pMUTIN-*floT-eyfp* |
| 5’FW20 | cctgaacattcaggttcaggttcaggtatg | Amplification of *eyfp* for Gibson Assembly of pMUTIN-*floT-eyfp* |
| 5’FW23 | gtgagcgctcacaattaatgatgataatcgtcatgttac | Amplification of *plsY* for Gibson Assembly of pAISH-*plsY-gfp* |
| 3’FW43 | cctgaacctgaacctgacatccattttattttaggttcttc | Amplification of *plsY* for Gibson Assembly of pAISH-*plsY-gfp* |
| 5’FW44 | aaatggatg*tcaggttcaggttcaggt*atggctagcaaaggagaag | Amplification of *gfp* for Gibson Assembly of pAISH-*plsY-gfp* |
| 3’FW44 | gtattacatatgtaagatttttatttgtagagctcatccatg | Amplification of *gfp* for Gibson Assembly of pAISH-*plsY-gfp* |
| 5’FW65 | aaa*gaattc*ttaggaggaaattattgaatgatttatgcatttatagg | Amplification of *cydB* for construction of pWhiteWalker 7 (*cydB*-*mCherry+plsY-gfp*), extends *cydB* with an EcoRI restriction enzyme site (italic) |
| 3’FW65 | ttt*gctagc*tgatttctttccttcaacata | Amplification of *cydB* for construction of pWhiteWalker 7 (*cydB*-*mCherry+plsY-gfp*), extends *cydB* with an NheI restriction enzyme site (italic) |
| 5’FW66 | aaagaattcttaggaggaaattattgaatgaatattccgaaccagattacgg | Amplification of *pgsA* for construction of pWhiteWalker 8 (*pgsA*-*mCherry+plsY-gfp*), extends *pgsA* with an EcorI restriction enzyme site (italic) |
| 3’FW66 | aaagctagctttttgtttaaaaacatctctacc | Amplification of *pgsA* for construction of pWhiteWalker 8 (*pgsA*-*mCherry+plsY-gfp*), extends *pgsA* with an NheI restriction enzyme site (italic) |
| 5’FW112 | taactttaagaaggagacaataatgcgtaccctgtattatttc | Amplification of *mreD* as a template and further for Gibson Assembly of pWALDO-*mreD-eyfp* |
| 3’FW112 | gaattgaccctggaagtacaggttttcccactgacgacgtttcatatcg | Amplification of *mreD* as a template and further for Gibson Assembly of pWALDO-*mreD-eyfp* |
| 3’FW113 | gaattgaccctggaagtacaggttttcaccgctgccgctaccgctccactgacgacgtttcatatcgatgtcg | Amplification of *mreD* as a template and further for Gibson Assembly of pWALDO-*mreD-eyfp* |
| 3’FW114 | ctcaccatgaattgaccctggaagtac | Amplification of *mreD* as a template and further for Gibson Assembly of pWALDO-*mreD-eyfp* |
| 5’FW115 | tggtgatgatgatgggccgcatttttatacagttcatccatacc | Amplification of *eyfp* for Gibson Assembly of pWALDO-*mreD-eyfp* |
| 3’FW115 | ggtcaattcatggtgagcaaaggtgaag | Amplification of *eyfp* for Gibson Assembly of pWALDO-*mreD-eyfp* |
|  |  |  |
| 5’FW116 | ctagagtcgagggtacaggaggtgtaatatttatgatgataatc | Amplification of *plsY* for Gibson Assembly of pMUTIN-*plsY-eyfp* and pMUTIN-*plsY-meyfp* |
| 3’FW116 | gaacctgacatccattttattttaggttcttc | Amplification of *plsY* for Gibson Assembly of pMUTIN-*plsY-eyfp* and pMUTIN-*plsY-meyfp* |
| 5’FW117 | aaatggatgtcaggttcaggttcaggtatg | Amplification of *eyfp* for Gibson Assembly of pMUTIN-*plsY-eyfp* and pMUTIN-*plsY-meyfp* |
| 3’FW117 | attaggcgggctgcattacttgtacagctcgtc | Amplification of *eyfp* for Gibson Assembly of pMUTIN-*pgsA-eyfp*, pMUTIN-*floT-eyfp*, pMUTIN-*plsY-eyfp* and pMUTIN-*plsY-meyfp* |

Nucleotide sequences in italic indicate added nucleotides to the original template sequence or introduced restriction enzyme sites if primers were used for non-gibson assembly cloning. Underlined nucleotides indicate homologues sequences to the template.

**Bacterial strain and plasmid construction**

Construction of PlsY/PgsA/FloT eYFP and PlsY-meYFP fusions in *S. aureus* SH1000:

In order to construct pMUTIN derivatives containing a fusion of *plsY*, *pgsA* or *floT* inframe with *eyfp*, *plsY* (5’FW116/3’FW116), *pgsA* (5’FW16/3’FW16) and *floT* (5’FW19/3’FW19), were amplified using SH1000 genomic DNA as a template and *eyfp* (for *plsY*: 5’FW117/3’FW117; for *pgsA*: 5’FW17/3’FW117; for *floT*: 5’FW20/3’FW117; were amplified using plasmid pKASBAR-*ezrA-eyfp* [^9^](#_ENREF_8) as a template. For the construction of a *plsY-meyfp* fusion in pMUTIN, *eyfp* was amplified from plasmid pKASBAR-*ezrA-meyfp*[^9^](#_ENREF_8). DNA fragments were cloned into the *KpnI/SpeI* site of pMUTIN-*gfp*+ using Gibson Assembly and transformed into *E. coli* NEB5α. Recombinant plasmids were tested by restriction digest and validated by DNA sequencing (GATC Biotech AG, Konstanz, Germany). The resulting plasmids were electroporated into RN4220 and from there transduced into SH1000 creating strains FW1 (*plsY-eyfp*), FW2 (*pgsA-eyfp*), FW8 (*floT-eyfp*) and FW10 (*plsY-meyfp*).

Construction of a Cls2-eYFP fusion in *S. aureus* SH1000:

The gene encoding for the major cardiolipin synthase *cls2* is likely to be organised in an operon since its downstream gene, *SAOUHSC_02324* (hypothetical protein), begins immediately after the stop codon of *cls2.* The use of pMUTIN could therefore affect the expression of *SAOUHSC_02324*. pKASBAR^8^ is a pCL84 derivative that is non-replicative in gram positive bacteria and integrates into the lipase gene *geh*^12^. This integration is facilitated by site specific recombination via help of an integrase between an *attB* site located within the lipase gene and an *attP* site located on pKASBAR. A promoter prediction suggests (http://www.fruitfly.org/) that the promoter of *cls2* is localised within 100 bp upstream of the start codon.

Single-copy expression of *cls2* under its native promoter was achieved via integration of a pKASBAR plasmid carrying *cls2* and its promoter into the genome of a *cls2* negative RN4220 strain. The *cls2* gene from RN4220 was knocked out by transduction of a JE2 NARSA transposon library[^13^](#_ENREF_12) strain containing a transposon at the beginning of *cls2* (JE2 NE258) resulting in RN4220 Δ*cls2::Tn*. In order to construct pKASBAR-*P_cls2_-cls2-eyfp*, *cls2* and its upstream sequence (5’FW09/3’FW09) were amplified from SH1000 genomic DNA, cloned into the *BamHI/AscI* site of pKASBAR-*ezrA-eyfp* using Gibson Assembly replacing *ezrA* by *P_cls2_-cls2*. The resulting plasmid was transformed into *E. coli* NEB5α. Recombinant plasmids were tested by restriction digest and validated by DNA sequencing (GATC Biotech AG, Konstanz, Germany). The resulting plasmid, pKASBAR-*P_cls2_-cls2-eyfp,* was electroporated into RN4220 expressing an integrase from plasmid pYL112Δ19 and from there transduced into RN4220 Δ*cls2::Tn* creating strain FW5.

Construction of an IPTG-inducible MreD-eYFP fusion in *E. coli* C43 (DE3)

In order to construct an IPTG-inducible fusion of *mreD-eyfp*, *mreD* (5’FW112/3’FW113) was amplified including a six amino acid linker sequence (SGSGSG) at the N-terminus of *mreD* using a codon-optimised *mreD* gene provided by GeneArt™ (Thermo Fisher Scientific, Waltham, United States of America) as a template. The PCR products were purified and served as a template for further PCR amplification (5’FW112/3’FW114). The gene encoding for *eyfp* was amplified with primers 5’FW115/3’FW115 using codon optimised linear DNA fragments provided by GeneArt™ (Thermo Fisher Scientific, Waltham, United States of America) as a template. All DNA fragments were gel purified and cloned into the *XhoI/HindIII* site of pWALDO-*gfp*-E[^6^](#_ENREF_5) using Gibson Assembly, followed by transformation into *E. coli* NEB5α. Recombinant plasmids were tested by restriction digest and DNA sequencing (GATC Biotech AG, Konstanz, Germany) and electroporated into *E. coli* C43 (DE3) creating strain *E. coli* C43 (DE) *mreD-eyfp*.

Construction of IPTG-inducible *plsY-gfp* with *cydB-mCherry* or *pgsA-mCherry* fusions:

In order to construct pWhiteWalker7 (*plsY-gfp* + *cydB-mCherry*) and pWhiteWalker8 (*plsY-gfp* + *pgsA-mCherry*), genes encoding for *cydB* and *pgsA* were amplified using primer pairs 5`FW65/3`FW65 and 5’FW66/3’FW66 respectively. DNA fragments were cut with *EcoRI* and *NheI* and ligated into pWhiteWalker3[^7^](#_ENREF_6) that was cut with the same enzymes replacing *mreD* with *cydB* or *pgsA*. Recombinant plasmids were tested by restriction digest and DNA sequencing (GATC Biotech AG, Konstanz, Germany), followed by electroporation into RN4220 creating strains FW16 (*plsY-gfp* + *cydB-mCherry*) and FW17 (*plsY-gfp* + *pgsA-mCherry*).

Construction PlsY-GFP fusions in CL, LPG and WTA deficient strains in *S. aureus* SH1000

In order to study PlsY-GFP localisation in a strain lacking cardiolipin, chromosomally integrated pMUTIN-*plsY-gfp* from JGL232 was transduced to SH1000 Δ*cls1*Δ*cls2*^11^, a strain lacking both known cardiolipin synthases in *S. aureus* creating strain FW21. Integration of pMUTIN-*plsY-gfp* at the native locus of *plsY* was confirmed via PCR.

Due to selection marker purposes, a tetracycline resistant version of pMUTIN-*plsY-gfp* was constructed using the pMUTIN derivative pAISH1^5^. This was achieved by *plsY* (5’FW23/3’FW43) amplification using SH1000 genomic DNA as a template and amplification of *gfp* (5’FW44/3’FW44) from plasmid pMUTIN-*gfp*+ [^4^](#_ENREF_3). DNA fragments were cloned into the HindIII/SwaI site of pAISH1 using Gibson Assembly and transformed into *E. coli* DC10B. Recombinant plasmids were tested by restriction digest with NheI and SacI and validated by DNA sequencing (GATC Biotech AG, Konstanz, Germany). The resulting plasmid, pAISH-*plsY-gfp*, was electroporated into RN4220 and from there transduced into SH1000 creating strain FW9. Genomic integration at the *plsY* locus was confirmed by PCR amplification.

PlsY-GFP localisation in a strain lacking LPG was achieved by replacing the gene *mprF* by an erythromycin resistance cassette by transduction of Δ*mprF::ermB* from strain SA113 Δ*mprF::ermB*^[13](#_ENREF_13)^ to FW9 creating FW22. The replacement of *mprF* by *ermB* and integration of pAISH-*plsY-gfp* was verified by PCR.

The knockout of WTAs was facilitated by transduction of Δ*tarO::ermB* from a complemented *tarO* mutant (SA113 Δ*tarO::ermB* + pRB‑tarO)[^1^](#_ENREF_14)^5^ to FW9 resulting in FW23. The replacement of *tarO* by *ermB* was verified by PCR.

Western blots of single-copy expressed GFP/eYFP fusions in *S. aureus* SH1000

Western blots were performed using isolated membrane fractions of *S. aureus* SH1000, FW1 (*plsY-eyfp*), JGL231 (*secY-gfp),* FW8 (*floT-eyfp*), FW5 (*cls2-eyfp*) and FW2 (*pgsA-eyfp*)*.* Strains were grown o/n in TSB supplemented with erythromycin (5 μg ml^-1^), lincomycin (25 μg ml^-1^) and IPTG (1 mM). O/n cultures were diluted to an OD_600_ of 0.05 in 1 l TSB and grown to an OD_600_ of 0.8-1. Cells were harvested, washed three times in ddH_2_O at 4°C, resuspended in TBSi (50 mM Tris, 100 mM NaCl, pH 8, plus protease inhibitor cocktail, Sigma-Aldrich) and lysed using a MP Fast Prep-24 (MP Biomedicals) (12 cycles for 30 s at 7 m/s). Samples were incubated for 3 min on ice between cycles. The membrane fraction of *S. aureus* was prepared as previously described^7^. Collected membranes were resuspended in PBS containing 1 % SDS (w/v).

Unspecific cross-reactivity of rabbit anti-GFP antibodies was removed as described by Sambrook and Russel^16^, except the antibodies were incubated with the whole cell lysate of SH1000 *spa::kan* o/n at 4°C. Anti-GFP antibodies at a dilution of 1:5,000 were used as a primary antibody and goat anti-rabbit-peroxidase antibodies (A0545, Sigma-Aldrich) at a dilution of 1:10,000 were used as a secondary antibody. The Western blot was developed using Clarity ECL Western Blotting Substrate (BioRad) followed by the manufacturer instructions.

Western blots of mCherry fusion constructs expressed from FRET plasmids in *S. aureus* RN4220

O/n cultures of strains *S. aureus* RN4220 FW13 (*gfp-mCherry* (tandem))*,* FW14 (*mreD-mCherry + plsY-gfp*), FW15 (*cdsA-mCherry + plsY-gfp*), FW16 (*cydB-mCherry + plsY-gfp*), FW17 (*pgsA-mCherry + plsY-gfp*), FW18 (*plsY-gfp*), FW19 (*secY-mCherry + plsY-gfp*) and FW20 (*mscL-mCherry + plsY-gfp*) were diluted in 50 ml TSB containing erythromycin (μg ml^-1^) and IPTG (0.5 mM) to an OD_600_ of 0.05 in 50 ml TSB and grown to an OD_600_ of 0.8-1. Harvested cells were washed three times in ddH_2_O at 4°C and resuspended in PBS supplemented with protease inhibitor cocktail (Sigma-Aldrich). Cells were lysed as described before and 10 ug of total protein was separated by gel-electrophoresis followed by estern blotting as described before. Western blots were performed using rabbit anti-mCherry antibodies (ab167453, Abcam) at a dilution of 1:5,000 as a primary antibody and goat anti-rabbit-peroxidase antibodies (A0545, Sigma) at a dilution of 1:10,000 as a secondary antibody.

Data Availability

All data generated or analysed during this study are included in this published article (and its Supplementary Information files).

1 Parsons, J. B. & Rock, C.O. Bacterial lipids: metabolism and membrane

homeostasis. *Progress in lipid research* **52**, 249-276 (2013).

2 Monk, I. R., Shah, I. M., Xu, M., Tan, M. W. & Foster, T. J. Transforming the untransformable: application of direct transformation to manipulate genetically *Staphylococcus aureus* and *Staphylococcus epidermidis*. *mBio* **3**, doi:10.1128/mBio.00277-11 (2012).

3 Wagner, S. *et al.* Tuning *Escherichia coli* for membrane protein overexpression. *Proceedings of the National Academy of Sciences of the United States of America* **105**, 14371-14376, doi:10.1073/pnas.0804090105 (2008).

4 Kaltwasser, M., Wiegert, T. & Schumann, W. Construction and application of epitope- and green fluorescent protein-tagging integration vectors for *Bacillus subtilis*. *Applied and environmental microbiology* **68**, 2624-2628 (2002).

5 Aish, J. L. Environmental regulation of virulence determinant expression in *Staphylococcus aureus*, University of Sheffield, (2003).

6 Waldo, G. S., Standish, B. M., Berendzen, J. & Terwilliger, T. C. Rapid protein-folding assay using green fluorescent protein. *Nature biotechnology* **17**, 691-695, doi:10.1038/10904 (1999).

7 Garcia-Lara, J. *et al.* Supramolecular structure in the membrane of *Staphylococcus aureus*. *Proceedings of the National Academy of Sciences of the United States of America* **112**, 15725-15730, doi:10.1073/pnas.1509557112 (2015).

8 Bottomley, A. L. *et al.* *Staphylococcus aureus* DivIB is a peptidoglycan-binding protein that is required for a morphological checkpoint in cell division. *Molecular microbiology*, doi:10.1111/mmi.12813 (2014).

9 Wacnik, K. Dissecting cell division in the human pathogen *Staphylococcus aureus*, PhD Thesis, University of Sheffield, (2016).

10 Horsburgh, M. J. *et al.* *sigmaB* modulates virulence determinant expression and stress resistance: characterization of a functional *rsbU* strain derived from *Staphylococcus aureus* 8325-4. *J Bacteriol* **184**, 5457-5467 (2002).

11 Tsai, M. *et al.* *Staphylococcus aureus* requires cardiolipin for survival under conditions of high salinity. *BMC microbiology* **11**, 13, doi:10.1186/1471-2180-11-13 (2011).

12 Lee, C. Y., Buranen, S. L. & Ye, Z. H. Construction of single-copy integration vectors for *Staphylococcus aureus*. *Gene* **103**, 101-105 (1991).

13 Fey, P. D. *et al.* A genetic resource for rapid and comprehensive phenotype screening of nonessential *Staphylococcus aureus* genes. *mBio* **4**, e00537-00512, doi:10.1128/mBio.00537-12 (2013).

14 Peschel, A. *et al.* *Staphylococcus aureus* resistance to human defensins and evasion of neutrophil killing via the novel virulence factor MprF is based on modification of membrane lipids with l-lysine. *The Journal of experimental medicine* **193**, 1067-1076 (2001).

15 Weidenmaier, C. *et al.* Role of teichoic acids in Staphylococcus aureus nasal colonization, a major risk factor in nosocomial infections. *Nat Med* 10(3):243-5, (2004).

# 16 [Sambrook J](https://www.ncbi.nlm.nih.gov/pubmed/?term=Sambrook%20J%5BAuthor%5D&cauthor=true&cauthor_uid=22485921) and [Russell DW](https://www.ncbi.nlm.nih.gov/pubmed/?term=Russell%20DW%5BAuthor%5D&cauthor=true&cauthor_uid=22485921). SDS-Polyacrylamide Gel Electrophoresis of Proteins. [*CSH Protoc.*](https://www.ncbi.nlm.nih.gov/pubmed/22485921) 2006(4) doi:10.1101/pdb.prot4540 (2006).

# 17 Rath *et al.* Detergent binding explains anomalous SDS-PAGE migration of membrane proteins. *Proceedings of the National Academy of Sciences of the United States of America* 106, 1760-1765, doi:10.1073/pnas.0813167106 (2008).

18 Nieto C and Espinosa M. Construction of the mobilizable plasmid pMV158GFP, a derivative of pMV158 that carries the gene encoding the green fluorescent protein.
*Plasmid*. 49(3):281-5 (2003).
